# Non-faradaic capacitive cation sensing under flow†

**DOI:** 10.1039/d4sc05271d

**Published:** 2024-09-09

**Authors:** Sophie C. Patrick, Robert Hein, Paul D. Beer, Jason J. Davis

**Affiliations:** a Department of Chemistry, University of Oxford South Parks Road Oxford OX1 3QZ UK jason.davis@chem.ox.ac.uk

## Abstract

The ability to continually monitor target ion species in real-time is a highly sought-after endeavour in the field of host–guest chemistry, given its direct pertinence to medical and environmental applications. Developing methodologies which support sensitive and continuous ion sensing in aqueous media, however, remains a challenge. Herein, we present a versatile and facile, proof-of-concept electrochemical sensing methodology based on non-faradaic capacitance, which can be operated continuously with high temporal resolution (≈1.4 s), in conjunction with custom-designed integrated microfluidics. The potential of this method is demonstrated for cation sensing at a chemically simple benzo-15-crown-5-based molecular film (B15C5_SAM_) as a representative redox-inactive, receptive interface. Detection limits as low as 4 μM are obtained for Na^+^ by these entirely reagentless analyses, and are additionally characterised by exceptional baseline stabilities that are able to support continuous sensing over multiple days. The platform performs well in artificial sweat across physiologically relevant spans of sodium concentration, and provides meaningful dose-dependent responses in freshwater samples. Finally, the high assay temporal resolution affords an ability to resolve both the kinetics of binding (association/dissociation) and notably characteristic fingerprints for different alkali metals which may be diagnostic of different interfacial ion binding modes.

## Abstract 2

The ability to continually monitor target ion species in real-time is a highly sought-after endeavour in the field of host–guest chemistry, given its direct pertinence to medical and environmental applications.

## Graphical abstract

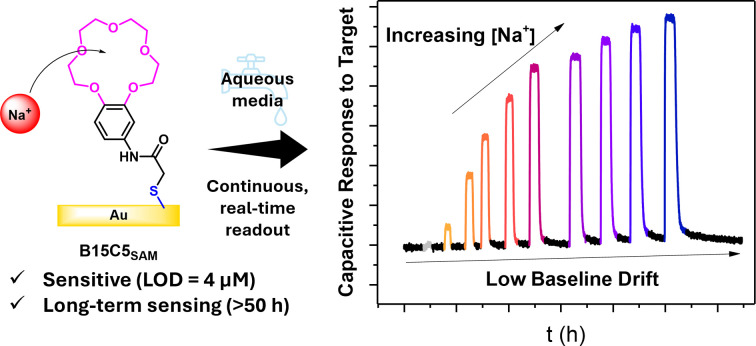

## Introduction

Sensing methodologies have developed rapidly in recent years to meet the demand for sensitive, selective, and quantitative target ion species assaying within a variety of environmental, industrial and medicinal scenarios.^1–4^ Electrochemical techniques constitute a significant number of these, owing to their associated high sensitivity, versatility and low cost,^2,5–7^ as well as the relative ease with which derived sensors can be miniaturised and integrated into microfluidic formats (promising high-throughput analyses with low required sample volumes, <1 mL).^5,8,9^ Within these, methodologies based on impedance-derived techniques have been shown to offer particularly sensitive and non-destructive routes to probe interfacial recognition, and can, in principle, be conducted in a continuous manner.^2,10–18^ This has been exemplified in recent work whereby specific redox capacitance responses at redox-active, ion-receptive interfaces have been shown to transduce anion recruitment in real-time.^10^ In this instance, the presence of an appended redox transducer engenders both a control of binding thermodynamics (through judicious application of the applied polarisation potential) and transduction. In such (faradaic) formats, however, there is an inevitable signal loss due to voltammetric degradation of the appended redox-unit, somewhat limiting the longevity of this approach to a few hours (up to 4 h reported thus far).^10^ In the absence of any film faradaic activity, capacitance spectroscopy can still sensitively report on both cation^19–22^ and anion^11^ recruitment at receptive interfaces.^2,11^ These reagentless interfacial capacitance analyses negate the synthetic requirement of introducing a redox-active unit into receptive motifs,^23–27^ and the baseline issues that typically result from its progressive degradation.^10,28^ To date this non-faradaic capacitance sensing approach has been limited to static solutions, without temporal analysis, and no real-world-relevant samples. By tracking ion binding events in real-time, valuable insight may be gained in resolving interfacial ion association/dissociation kinetics, about which little is known.

Herein, we present a proof-of-principle, reagentless sensing methodology based on non-faradaic capacitance spectroscopy to monitor changes in the interfacial capacitance of a model benzo-15-crown-5 interface upon cation binding. This enabled the continuous (>50 h), real-time flow detection of cations in water, including both artificial sweat and freshwater samples, with high temporal resolution (≈1.4 s) and high sensitivity (LOD(Na^+^) = 4 μM). Temporal analyses, furthermore, resolve kinetic signatures that are distinct for smaller and larger alkali metal cations, observations potentially indicative of unique interfacial binding modes.

## Results and discussion

### Synthesis of B15C5 & characterisation of derived receptive films

In this work, the well-established benzo-15-crown-5 (B15C5) motif (selective for alkali metal cations, particularly Na^+^, through size complementarity) was employed as a representative ion receptive binding unit,^29^ and immobilised onto gold sensor surfaces through an adjacent anchoring disulfide moiety.

**Scheme 1:**
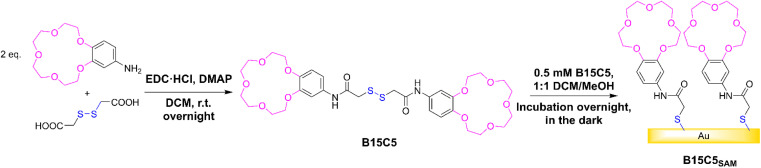
Reaction scheme of B15C5 synthesis and self-assembly into B15C5_SAM_.

The target receptor, B15C5 was synthesised in one step from a one-pot reaction between dithioglycolic acid and 4′-aminobenzo-15-crown-5, with 4-dimethylaminopyridine (DMAP) and 1-ethyl-3-(3-dimethylaminopropyl)carbodiimide hydrochloride (EDC·HCl), in anhydrous DCM at room temperature (see Scheme 1). Following purification, B15C5 was afforded in good yield and characterised by ^1^H and ^13^C NMR, and high-resolution mass spectrometry (see ESI, Fig. S2 and S3†). Self-assembled monolayers (SAMs) were formed *via* incubation of clean gold disc electrodes in 0.5 mM B15C5 in 1:1 DCM/MeOH overnight in the dark (Scheme 1, see ESI Section S3† for further detail) and extensively characterised (Table 1).

**Table 1:** Characterisation data for B15C5_SAM_. Errors were derived from three independent repeats

	Contact angle (°)	Film thickness (nm)	Surface coverage (×10^−10^ mol cm^−2^)	Static film capacitance (μF cm^−2^)	Film dielectric, *ε*_r_
B15C5_SAM_	62.1 ± 1.3	1.02 ± 0.2	1.23 ± 0.29	3.68 ± 0.18	4.24 ± 0.21

X-ray photoelectron spectroscopy (XPS) spectra were consistent with expectations (Fig. S4–S8, Tables S1 and S2†), revealing the presence of only C, N, O and S in the film and good agreement between the predicted and obtained atomic ratios based on the B15C5 chemical composition. Similarly, the ATR-FTIR spectrum of B15C5_SAM_ displayed the expected peaks: a broad amide N–H stretch at ≈3400 cm^−1^, aromatic C–H stretches at ≈2900 cm^−1^ and an amide carbonyl C=O stretch at 1649 cm^−1^ (Fig. S9†). Water contact angle measurements were representative of a predictably hydrophilic interface (62 ± 1°, consistent with previous reports of similar 15C5-based molecular films, *cf.*, 55 ± 1°).^19^ Film thicknesses of 1.02 ± 0.2 nm were determined through ellipsometry, which were supportive of densely-packed monolayers where the receptors are oriented “upright” on the Au surface. Thiol stripping resolved molecular densities (1.23 ± 0.29 × 10^−10^ mol cm^−2^, corresponding to a molecular footprint of 1.41 ± 0.33 nm), and capacitive Bode plot phase angles of ≈85° (see Fig. S10,†*cf.* ideal dielectric capacitor = 90°) were consistent with densely-packed film generation. The film dielectric of ≈4 (resolved *via* the film capacitance as described by eqn (1), see below) is also reflective of a high molecular density and significant solvent exclusion.^11^

### Non-faradaic capacitive ion sensing performance of B15C5_SAM_

**Fig. 1:**
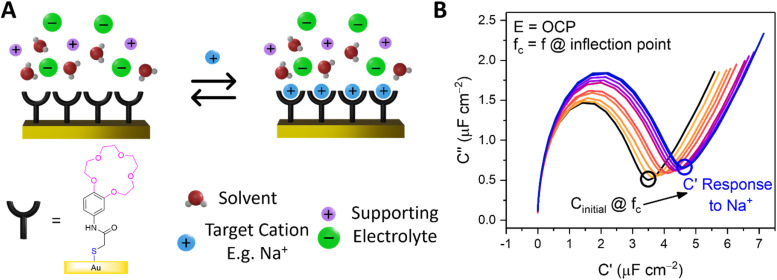
(A) Schematic depiction of B15C5_SAM_ in the absence (left) and presence (right) of a specific target. (B) Capacitive Nyquist plots of B15C5_SAM_ in H_2_O, 100 mM TEACl conducted at fixed OCP. Changes in *C* are measured at the inflection point of each Nyquist plot (corresponding to frequency, *f*_c_) upon addition of increasing concentrations of NaCl up to 50 mM (blue) in static solution.

Capacitive processes were probed at a fixed DC potential (the open-circuit potential, OCP), upon which a small amplitude AC perturbation (10 mV) was applied across a range of AC frequencies (1–100 000 Hz). The non-faradaic characteristics and resolved film capacitance, *C* (defined as the real component at the inflection point of the resulting capacitive Nyquist plot, see Fig. 1B) of B15C5_SAM_ can most simply be described by a plate capacitor Helmholtz model (eqn (1), where *ε*_r_ is film dielectric, *ε*_0_ is the permittivity of free space, *A* is electrode surface area, and *d* is film thickness).1
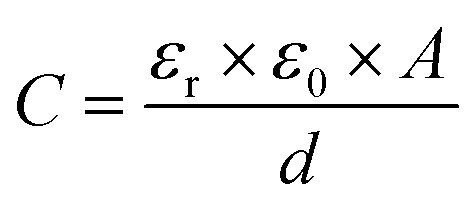


Assuming that there are no significant film rearrangements induced by target recruitment (*i.e.*, *d* remains constant), *C* is therefore directly proportional to the dielectric constant of the film, *ε*_r_. Predictably, then, ion recruitment to the receptive molecular film (*e.g.*, Na^+^ binding to B15C5_SAM_) and any concomitant enhanced layer hydration,^11^ results in an increase in film dielectric constant and a simultaneous increase in the value of *C* (see Fig. 1A).^11,12,30^ This well-established dependence has been borne out in a number of previous studies for non-faradaic ion sensing for both cations^19–22^ and, more recently, anions.^11,12^ A more detailed consideration of the individual capacitive components which contribute to this overall film capacitance, *C* can be found in the ESI, Section S4, Fig. S11 and S12.† As expected, then, exposing B15C5_SAM_ to increasing concentrations of NaCl in water results in progressive shifts in the inflection point of the capacitive Nyquist plots shown in Fig. 1B (that is, a growth in film capacitance).

### Static non-faradaic ion sensing

Unless stated otherwise, all cation sensing experiments were conducted in deionised H_2_O with 100 mM TEACl as supporting electrolyte. TEACl was chosen as the supporting electrolyte due to the non-coordinating nature of the organic cation (ionic radius of TEA^+^ = 4.5 Å, *cf.* 15C5 cavity diameter = 1.84 Å),^29,31^ meaning that no corrections for non-specific association with the crown film were required. In line with expectations, significant responses were observed with respect to the baseline *C* in the presence of target alkali metal cations in static solution (39 ± 5% modulation, Δ*C* = 1.7 ± 0.3 μF cm^−2^ at 50 mM Na^+^, see ESI Section S5, Fig. S13†).^19–21^

In contrast, negligible responses (Δ*C*_max_ = 0.007 μF cm^−2^, *C*_rel_ = 1.2%) were observed upon exposure of equivalent cation loadings to a non-receptive 1-dodecanethiol interface (see ESI Fig. S14†), confirming the requirement of specific ion recognition to induce a measurable response.

**Fig. 2:**
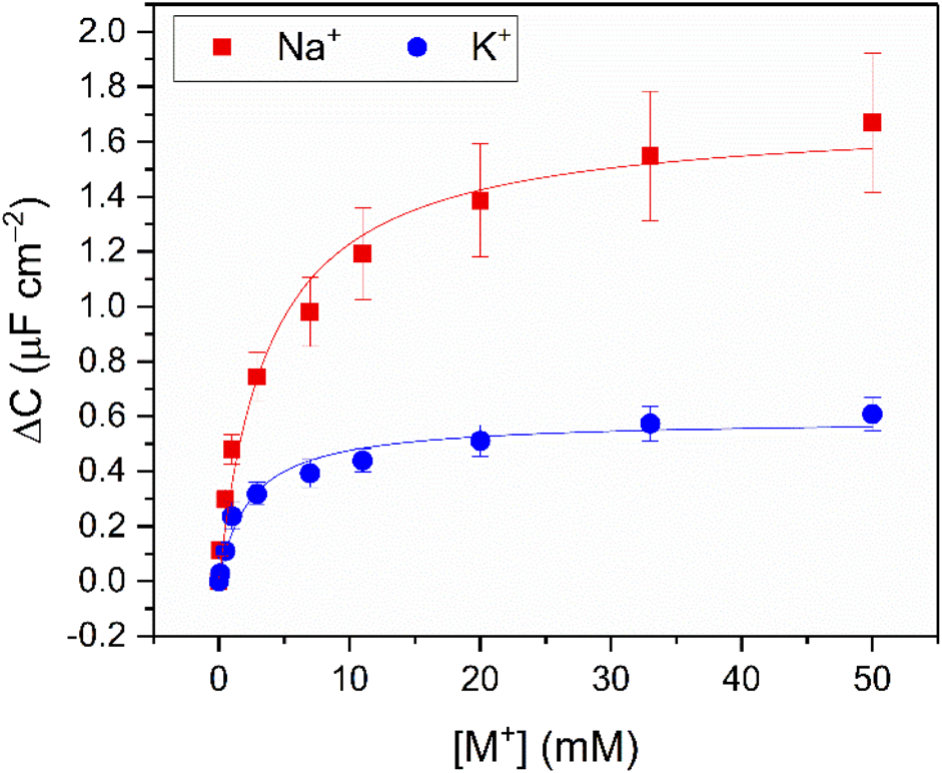
Response isotherms of B15C5_SAM_ determined from the shift in *C*′ in response to increasing concentrations of Na^+^ (red squares) and K^+^ (blue circles) in H_2_O with 100 mM TEACl as supporting electrolyte, under static conditions. Isotherms were fitted to a Langmuir model (see ESI Section S1.6 for further details, and eqn (S1)†). Errors represent one standard deviation of three independent repeats.

As expected,^19,21^ the resolved selectivity trend of the B15C5_SAM_ interface correlated with cation ionic size (see Fig. 4B, blue columns), with the largest responses observed upon addition of Na^+^, attributable to the excellent size complementarity of 15-crown-5 for Na^+^.^19–21,29^ Systematic sensing studies were performed for Na^+^ and K^+^ as target cations of interest (see Fig. 2), affording a sodium assay detection limit of 12.3 ± 3.6 μM and sensitivity of 0.44 ± 0.06 μF cm^−2^ mM^−1^. Additional data analysis and binding parameters for K^+^ can be found in the ESI (see ESI Section S5, Table S3†).

Importantly, these results highlight the high sensitivity of non-faradaic capacitance in transducing specific interfacial binding events. A translation to a continuous measurement modality is, of course relevant to downstream applications in environmental or healthcare monitoring applications. To this end, we describe below the development of capacitive assays, at fixed frequency and potential, in a flow cell. The resulting platform supports a facile, continuous sensory readout such that cation levels can be followed in real-time (see below).

### Continuous flow non-faradaic capacitive ion sensing

**Fig. 3:**
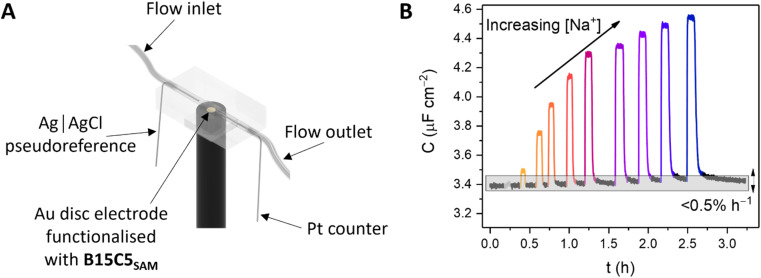
(A) Schematic depiction of the custom-designed, 3D-printed flow cell and electrochemical cell components. (B) Non-faradaic capacitance response, *C* of B15C5_SAM_ toward Na^+^ under continuous electrolyte flow (H_2_O, 100 mM TEACl) in the custom flow cell (cell volume = 10 μL, flow rate *Q* = 100 μL min^−1^, at fixed *E* = OCP and *f* = *f*_c_). Each spike represents the response toward aliquots (*V*_sample_ = 1 mL) of Na^+^ of concentration increasing up to 50 mM. Subsequent washing of the interface with supporting electrolyte re-establishes the baseline (black lines) with excellent recovery (0.5 ± 0.3% h^−1^).

Continuous flow ion sensing assays were enabled with the generation of a custom 3D-printed microfluidic chip, modelled on previous designs (but with a pseudoreference electrode – ESI, Section S6†)^10,28^ to house a standard 3-electrode electrochemical cell: Pt wire counter electrode, Ag|AgCl wire pseudoreference electrode and a receptor-modified Au disc working electrode (see Fig. 3A and S15, and ESI Section S6† for more details). This flow cell was combined with a sample injector to introduce aliquots of defined volume (typically 1 mL), and a syringe pump to continuously drive electrolyte through the flow system (100 mM TEACl in H_2_O). Prior to each measurement, the OCP was determined (typically 0–0.1 V *vs.* Ag|AgCl) and applied as the DC bias potential to acquire a capacitive Nyquist plot, from which *f*_c_ was determined. Repeat non-faradaic capacitance measurements were then performed at these fixed OCP and *f*_c_ values to afford a continual readout of *C* in real-time with high (≈1.4 s) temporal resolution. Introduction of sample cation aliquots induces significant “spikes” in the resulting sensogram (see Fig. 3B), immediately followed by a “washing” step whereby electrolyte is continuously flushed over the receptive interface to (re)establish the baseline (see black lines in Fig. 3B).

**Fig. 4:**
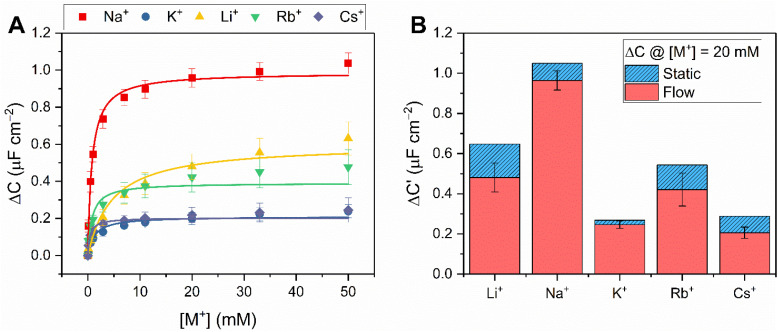
(A) Response isotherms determined from the shift in *C*′ in response to increasing concentrations of Na^+^ (red squares), K^+^ (blue circles), Li^+^ (yellow triangles), Rb^+^ (green inverted triangles) and Cs^+^ (purple diamonds) in H_2_O with 100 mM TEACl as supporting electrolyte, under continuous flow at 100 μL min^−1^. Isotherms were fitted to a Langmuir model (see ESI Section S1.6 for further details, and eqn (S1)†). Errors represent one standard deviation of three independent repeats. (B) Selectivity studies with B15C5_SAM_ under continuous flow (red) and static (blue, dashed) conditions upon addition of 20 mM of Li^+^, Na^+^, K^+^, Rb^+^ and Cs^+^ (in order of increasing ionic diameter ranging from 1.44–3.34 Å, *cf.* 15C5 cavity diameter = 1.84 Å).‡‡The disparity between the responses measured under static (red bars) and continuous flow (blue, dashed bars) conditions was attributed to the fact that non-specific (baseline) drifts can be more reliably identified and accounted for under continuous flow, real-time analyses without potentially incomplete/disruptive electrode removal and washing.^29^

The fidelity of baseline recovery is striking, with an almost negligible drift of Δ*C* = 0.03 ± 0.04 μF cm^−2^ h^−1^ (0.5 ± 0.3% h^−1^, errors correspond to 10 independent repeats across 3 different electrodes, over measurement timeframes ranging from 5 to 54 h) throughout titrations of a range of relevant cations (Li^+^, Na^+^, K^+^, Rb^+^, Cs^+^ or NH_4_^+^, see Fig. 4A).

Corroborating the observations made under static conditions (*vide supra*), the addition of the alkali metals (Li^+^, Na^+^, K^+^, Rb^+^ and Cs^+^) and NH_4_^+^ under continuous flow induced significant changes in *C* (up to Δ*C* = 1.1 ± 0.1 μF cm^−2^, or 33%, see Fig. 4A, S16 and S17†). Fig. 4B contrasts the response of B15C5_SAM_ to each cation at [M^+^] = 20 mM under static and continuous flow conditions, highlighting the consistency in resolved selectivity trends and the clear preference of B15C5_SAM_ for Na^+^ in both cases. Expectedly, the selectivity trend of B15C5_SAM_ for the alkali metals based on the maximum *C* response to each cation generally reflects the trends previously reported for cation binding at 15C5-based hosts in solution (K = Na^+^ > K^+^ > Li^+^ > Cs^+^ in ACN).^32^ However, the resolved binding constants for B15C5_SAM_ (K = Cs^+^ > Na^+^ > Rb^+^ > K^+^ > Li^+^, tabulated in Table S4 in the ESI†), deviate slightly from this trend in that larger cations such as Rb^+^ and Cs^+^ bind more strongly than expected (*K* = 763 ± 197 M^−1^ and 1929 ± 640 M^−1^ in H_2_O, respectively), potentially due to 2:1 host–guest sandwich complex formation. The formation of such 2:1 sandwich complexes also inevitably has kinetic implications for binding, something which is explored later (see below).

Once again, negligible changes in *C* (Δ*C* < 1% in all cases) upon injection of either “blank” samples of 100 mM TEACl at B15C5_SAM_, or mM target cation levels at a non-receptive 1-dodecanethiol interface were observed (Δ*C* < 2%, see Fig. S18†). Furthermore, markedly attenuated responses were observed during competition studies when either Na^+^ or K^+^ were precomplexed with excess [2.2.2]-cryptand (K(Na^+^) = 8000 M^−1^ in H_2_O)^33^ compared to the responses of B15C5_SAM_ to Na^+^ or K^+^ alone (see Fig. S19 and S20†), in line with expectations for competitive recruitment.

Systematic sensing studies were then conducted for all cations of interest under continuous flow by titrating in increasing concentrations of MCl salts (at a constant ionic strength of 100 mM to mitigate non-specific electrolyte effects on *C* response; see Fig. S16† for representative sensograms for all target cations, and Fig. 4A & S17† for the corresponding response isotherms).

Derived response isotherms afforded a range of binding and analytical parameters, including the apparent binding constants, maximum response in *C*, sensitivity and LOD which are summarised for Na^+^ and K^+^ as representative target cations in Table 2, and Tables S4 and S5† for all other target cations.

**Table 2:** Analytical parameters and binding constants of B15C5_SAM_ of Na^+^ and K^+^ under continuous flow at 100 μL min^−1^ from Langmuir fitting (eqn (S1)) of response isotherms^a^

Target cation	Δ*C*_max_ (μF cm^−2^)	*K* (M^−1^)	Sensitivity (μF cm^−2^ mM^−1^)	LOD (μM)
Na^+^	1.03 ± 0.06	1252 ± 341	0.52 ± 0.05	4.0 ± 1.2
K^+^	0.29 ± 0.02	555 ± 58	0.09 ± 0.01	27.4 ± 4.7

a Errors represent one standard deviation of at least three independent repeats. The interface sensitivity was obtained by analysis of the pseudolinear region at low concentration (0–1 mM [M^+^]), which was then used to calculate the limit of detection (LOD). Further details on these calculations are included in the ESI Section S1.8.

As is apparent from the acquired data, and discussions thus far, the crown SAM sensor is persistently most sensitive to Na^+^, demonstrating a (high) sensitivity of 0.52 ± 0.05 μF cm^−2^ mM^−1^ under continuous flow, corresponding to a highly competitive limit of detection of 4.0 ± 1.2 μM. This exceeds that typical of most commercial ion selective electrodes (the industry standard for in-the-field Na^+^ sensing).^34,35^ Notably, this level of sensitivity is achieved with a simple, single-component model interface (*cf.* standard ISEs require a suitable ionophore, ion-exchanger and polymer matrix),^36^ and does not require re-calibration during measurements. Additionally, since data acquisition is at OCP nor any chosen potential, the method presented herein is insensitive to reference electrode potential drift (which is often problematic for ISEs).^37^

**Fig. 5:**
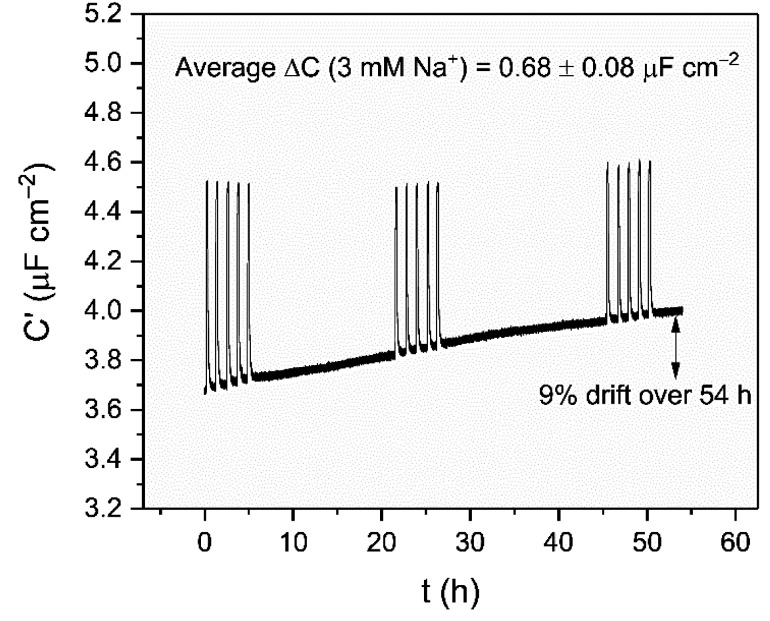
Long term cation sensing experiment conducted over 54 h in water (100 mM TEACl as supporting electrolyte). Responses of B15C5_SAM_ to 3 mM Na^+^ are shown as spikes in the sensogram.

Supported by the previously noted high baseline stability of the crown interface, the long-term sensing capability was then assessed by continually monitoring responses to repeat 3 mM Na^+^ injections into water over the course of 54 h (see Fig. 5). These were highly reproducible, with an average response of Δ*C* = 0.68 ± 0.08 μF cm^−2^ (*C*_rel_ = 17.9 ± 2.7%) across 15 additions. It should be noted that the impressive baseline stabilities mentioned earlier still persist over this entire timeframe (0.2% h^−1^), fully consistent with the shorter-term measurements (*cf.* 0.5 ± 0.3% h^−1^). Notably, this 54 hour sensing was performed in one continuous, undisturbed measurement, without re-conditioning or calibration; this is in stark contrast to the majority of commercial ion-selective electrodes which typically require re-calibration after a few hours in order to achieve their stated sensitivity and accuracy.^35,38^

**Fig. 6:**
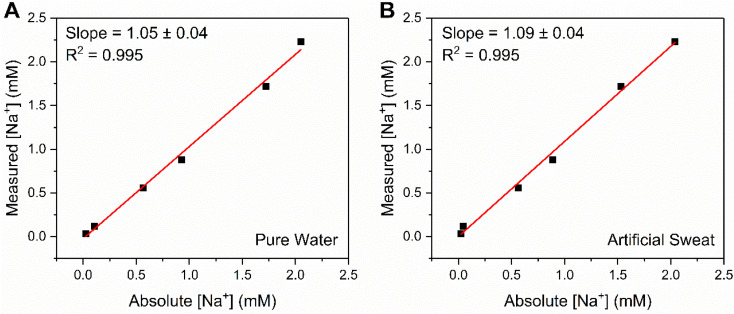
Correlation plot showing the excellent agreement between absolute and measured [Na^+^] values at B15C5_SAM_, for a range of unknown concentrations (up to 3 mM) in (A) pure water (100 mM TEACl) and (B) a simple, artificial sweat matrix (100 mM TEACl, 11.3 mM lactic acid, 21.6 mM urea in pure water). Measured [Na^+^] values were estimated from fitting a Langmuir–Freundlich model (eqn (S2)†) to a prior obtained calibration curve.

In challenging the crown capacitor interfaces with a range of blinded samples, excellent agreement (correlation slopes of 1.05 and 1.09 for pure water and artificial sweat, respectively) was observed between absolute and measured [Na^+^] values (6 samples between 0.03–3 mM Na^+^) in both pure (deionised) water (Fig. 6A) and a simple artificial sweat matrix (11.3 mM lactic acid, 21.6 mM urea in pure water, Fig. 6B). Three additional samples (9.47, 15.38 and 42.91 mM) were analysed in the artificial sweat matrix, corresponding to physiologically-relevant concentrations of Na^+^ (typically between 10–100 mM, see ESI Fig. S22†).^39^ The correlation between absolute and measured [Na^+^] in both cases was determined through cross reference to a prior acquired calibration curve (Fig. S21 and S22,† respectively). Further studies were then conducted in freshwater (Fig. S23†) and tap water (Fig. S24†) samples, wherein significant, stable, and dose-dependent responses to [Na^+^] (0–50 mM) were retained, demonstrating the potential of this methodology for target ion monitoring in real-world-relevant media.

### Temporal resolution of cation-dependent binding modes

**Fig. 7:**
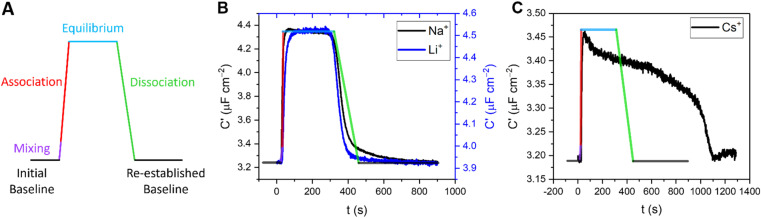
(A) Representative response signature expected under continuous flow for specific binding at a receptive interface, representing the resolved capacitance prior to target injection (baseline, black lines), and following injection which comprises a short mixing period (purple), association (red) and subsequent dissociation upon washing (green). This predicted signature is superimposed (with consistent scaling with respect to time) onto the measured responses of B15C5_SAM_ to 50 mM (B) Li^+^ (blue) and Na^+^ (black) and (C) Cs^+^ in water, with 100 mM TEACl as supporting electrolyte. It should be noted that all titration studies were performed in an identical and systematic manner for each of the cations, precluding physical or experimental disparities from inducing this different behaviour.

In addition to enabling cation quantification, the continuous sensing modality described above can resolve both association and dissociation regimes of binding; such kinetic analyses are rare in small molecule host–guest recognition (and virtually unknown at interfaces). Herein, we resolve two distinct temporal fingerprints that correlate with ionic size; smaller cations (Li^+^ and Na^+^) exclusively exhibit a rapid and reversible binding equilibration with a sharp return to baseline on decomplexation. For larger cations (K^+^, Rb^+^, Cs^+^ and NH_4_^+^, to an extent), an initial capacitative response is observed but is not stable and apparent decomplexation is slow, see Fig. 7 and S25–S30.†

In order to quantify this behaviour, the rates of the association (upon injection) and dissociation (upon washing) regimes of 5 concentrations of representative target cations (Li^+^, Na^+^, K^+^ and Cs^+^) were approximated by fitting a linear model to the first 10 data points. The relative flow rate to cell volume was such that kinetic analyses were free from any potential solution mixing artefacts (see ESI Section S8† for further information). Specific association and dissociation traces for selected cation concentrations can be found in the ESI Section S8, Fig. S25–S30, Tables S6 and S7.†

Interestingly, the association response rate constants for each of the cations appear to be broadly consistent with slightly higher rates observed for Na^+^, in good agreement with its binding affinity (*e.g.*, at 11 mM [M^+^] the association response rates for Li^+^, Na^+^, K^+^ and Cs^+^ were 17, 52, 9 and 11 nF cm^−2^ s^−1^, respectively). The approximated dissociation response rate constants, however, differ substantially, with Li^+^ and Na^+^ exhibiting broadly similar rates, but with significantly slower rates observed for K^+^ and particularly Cs^+^ (*e.g.*, at 11 mM [M^+^] the calculated dissociation response rates for Li^+^, Na^+^, K^+^ and Cs^+^ were 7, 8, 2 and 0.2 nF cm^−2^ s^−1^, respectively).

Although a direct chemical interpretation of these dissociation response rate regimes is challenging, we hypothesise that they report on different ion binding modes. Specifically, the significantly slower dissociation kinetics observed here for the larger cations (K^+^, Rb^+^ and NH_4_^+^, see ESI Fig. S27–S30†) may result from a 2:1 host–guest binding stoichiometry at the surface (schematically depicted in Fig. S31†).^19,21^ 15-Crown-5-based receptors are known to form stable intermolecular sandwich complexes with larger alkali metals (K^+^, Rb^+^ and Cs^+^) both in solution^40,41^ and at surfaces.§§Attempts to prevent 2:1 host–guest sandwich complex formation by generating mixed SAMs with an alkane thiol diluent (to increase the spatial separation between each B15C5 unit) were unfortunately unsuccessful.^19,21,42^

## Conclusions

A proof-of-principle, reagentless ion sensing methodology based on non-faradaic capacitance for continuous, real-time cation sensing at a benzo-15-crown-5 receptive molecular film is reported. The low baseline drift of these interfaces supports highly sensitive (LODs as low as 4 μM for Na^+^) and reagentless cation detection under flow across extended periods of time. Analyses conducted in real world media, including sweat, tap and fresh water, marks this work as a step change towards ion monitoring over practically-relevant time scales for environmental monitoring, for example. The temporal resolutions support the acquisition of previously inaccessible kinetic signatures of ion binding and unbinding. Though exemplified here with benzo-15-crown-5 molecular films, the methodology is generic and likely to be extendable to sensing of other (ionic) analytes at a wide range of redox-inactive receptive interfaces.

## Author contributions

S. C. P. performed all electrochemical ion sensing studies and acquired most of the surface characterisation data for B15C5_SAM_. R. H. synthesised and characterised B15C5 in solution and analysed XPS measurements. The project was conceptualised by R. H. and J. J. D. The paper was written by S. C. P., R. H., P. D. B. and J. J. D., with contributions from all authors.

## Conflicts of interest

There are no conflicts to declare.

## Supplementary Material

SC-015-D4SC05271D-s001

## Data Availability

The data supporting this article have been included as part of the ESI.†
