# Supplementary material for: Non-faradaic capacitive cation sensing under flow
Source: Chem Sci. 2024 Sep 9;15(44):18310–7. doi: 10.1039/d4sc05271d (PMC11382808; doi:10.1039/d4sc05271d)
Supplement: SC-015-D4SC05271D-s001 [file SC-015-D4SC05271D-s001.pdf]

Supplementary Information (SI) for Chemical Science.
This journal is © The Royal Society of Chemistry 2024

Supplementary Information for

Non-Faradaic Capacitive Cation Sensing Under Flow

Sophie C. Patrick[a], Robert Hein[a], Paul D. Beer[a], Jason J. Davis*[a]

[a]Department of Chemistry, University of Oxford, South Parks Road, Oxford OX1 3QZ, U.K.

Email: jason.davis@chem.ox.ac.uk

Table of Contents

S1.1: General Information 2
S1.2 XPS 2
S1.3: Electrochemical Measurements 2
S1.4: Electrode Pre-treatment and SAM Formation 2
S1.5: Capacitive Measurements 3
S1.6: Continuous Capacitive Measurements: Continuous Flow Set-Up 3
S1.7: Binding Isotherm Analysis 4
S1.8: Limit of Detection (LOD) and Sensitivity Determination 4
S1.9: Preparation of Real-world Water Samples 4
S2: Synthesis and Characterisation of B15C5 5
S3: Film Formation and Characterisation of B15C5$_{SAM}$ 7
S4 Capacitive Characteristics of Receptive Interfaces 12
S5 Static Cation Sensing Studies 15
S6 Microfluidic Flow Setup 17
S7 Real-time Ion Sensing Under Continuous Flow 19
S8 Resolving Different Binding Modes 25
S9 References 32

S1 Materials, Instruments and Methods

S1.1: General Information

All experiments were performed at room temperature in the presence of oxygen. All commercially available chemicals and solvents were used as received without further purification. Ultrapure water was obtained from a Milli-Q system (18.2 MΩcm).

S1.2 XPS

Samples were analysed using a Thermo Scientific K-Alpha XPS instrument equipped with a microfocussed monochromated Al X-ray source. The source was operated at 12 keV with the use of a 400-micron spot size. The analyser operates at a constant analyser energy S3 (CAE) of 200 eV for survey scans and 50 eV for detailed scans. Charge neutralisation was applied using a combined low energy electron/ion flood source.

S1.3: Electrochemical Measurements

All experiments were conducted using a PalmSens4 Potentiostat (PSTrace 5.9) with a three-electrode setup equipped with a gold disc working electrode (BaSi, 1.6 mm diameter) and platinum wire counter electrode. A Ag|AgCl wire pseudo reference electrode was utilised for all experiments, unless otherwise stated. All potentials reported are wrt. to this Ag|AgCl pseudo reference electrode. In all cases the ionic strength was maintained at a constant 100 mM throughout.

Unless stated otherwise, all cation sensing experiments were conducted in deionised H_2O with 100 mM TEACl as supporting electrolyte. TEACl was chosen as the supporting electrolyte due to the non-coordinating nature of TEA^+ ions (no specific binding predicted due to size constraints, ionic radius of TEA^+ = 4.5 Å, cf. 15C5 cavity diameter = 1.84 Å).[1, 2] Note that this concentration is consistent with the background ionic strength of a number of real aqueous samples of interest, including seawater (salinity ≈0.6 M)[3] or sweat (salinity ≈10-100 mM),[4] and that any other electrolyte could feasibly be used once electrolyte-film interactions had been defined.

S1.4: Electrode Pre-treatment and SAM Formation

Au disc electrodes were cleaned according to previously reported protocols.[5] Immediately following the cleaning procedure, the Au disc electrodes were rinsed thoroughly with water and ethanol and immersed in a solution of 0.5 mM **B15C5** in 1:1 DCM/MeOH overnight in the dark. Subsequently, the Au disc electrodes were rinsed with copious amounts of DCM and MeOH, then used immediately.

S1.5: Capacitive Measurements

The capacitive properties of the interface were obtained by electrochemical impedance spectroscopy (EIS).[6] Capacitive Nyquist plots were measured between 100 kHz and 1 Hz over 40 frequencies (logarithmically stepped), using a 10 mV AC amplitude and 5 s of equilibration time. The DC potential applied was the open circuit potential (OCP), which was acquired over 120 s with a stability criterion of 0.001 mV s^{-1}. The film capacitance, C was determined from the diameter of the semicircle of the capacitive Nyquist plot (approximated as C' at the inflection point), and the corresponding frequency at this capacitance was denoted as f_{film} (typically 80-150 Hz).

During static cation sensing studies, the 3-electrode cell described above was submerged in 4 mL of 100 mM TEACl in H_2O in a glass vial. For titrations, aliquots of 100 mM of the target cation salts, MCl (*e.g.*, 100 mM NaCl) were progressively added to the static cell, up to 50 mM [M^+]. The glass vial was swirled in between each addition to ensure effective mixing between the supporting electrolyte and analyte solution before each measurement. For selectivity studies, the **B15C5$_{SAM}$**-functionalised working electrode was removed from solution following each measurement of each target analyte solution (i.e., 20 mM of MCl where M^+ = Li^+, Na^+, K^+, Rb^+, Cs^+ or NH_4^+), rinsed with copious quantities of deionised water, then resubmerged in fresh electrolyte and the baseline *C* restabilised for at least 1 h prior to each addition.

Continuous capacitive measurements were performed with a time scan at fixed potential (OCP ascertained following the stabilisation procedure of repeat cycling of capacitive Nyquist plots, see below) and fixed frequency (f_{film}, unless otherwise stated). Prior to all cation sensing studies, a stabilisation procedure was performed under continuous flow at a flow rate of 100 µL min^{-1}, corresponding to the flow rate used for all sensing studies, unless otherwise stated. This involved repeat cycling of capacitive Nyquist plots for ≈1 h (resolved over a frequency range of 1-10,000 Hz at open-circuit potential, OCP) until a stable C' signal was obtained (see

below) and the determined OCP deviated by < 2 mV over 5 repeats. A stable C′ signal is defined here by a deviation of < 0.2 nF cm^{-2} of C′ at the inflection point of the capacitive Nyquist plot, which correlates to C_{rel} of ≈0.1% and can thus be considered a negligible drift. Continuous measurements were then conducted at fixed frequency (determined from the frequency at the C′ at the inflection point of the stabilised Nyquist plots, typically 75-150 Hz) and fixed potential (the stable OCP determined following stabilisation, ≈ 0.1 V). The integrated injector system (see Section S1.6) injected analyte solutions (of identical overall ionic strength, 100 mM) into the continuous flow line, inducing significant response "spikes" in the resulting sensogram (see Figure 3B, for example). Once a stable response was obtained (typically ≈ 4 min), the injector system was then switched back to a pure electrolyte line, enabling a "washing" step whereby electrolyte is continuously flushed over the receptive interface to (re)establish the baseline (see black lines in Figure 3B). Importantly, the re-establishment of the baseline through this washing step occurs seamlessly without removing the **B15C5$_{SAM}$**-functionalised electrode from solution at any point; in direct contrast to the static sensing studies described above.

S1.6: Continuous Capacitive Measurements: Continuous Flow Set-Up

The continuous flow setup is detailed in previously reported work[7, 8] with the exception of an adapted 3D-printed microfluidic cell (see Section S6, Figure S15 for the blueprint of the new flow cell), and is summarised here. The set-up comprises the adapted 3D-printed microfluidic cell, a syringe pump to continuously push electrolyte through the setup at a flow rate of 100 μL min^{-1} (unless otherwise stated) and an integrated injector system through which the analyte solutions (of identical overall ionic strength) were injected into the continuous flow line. As in previous studies, the majority of tubing used was Cole-Parmer PEEK tubing other than a small connecting section from the main tubing line to the cell, which was Cole-Parmer MasterFlex Peristaltic tubing.

S1.7: Binding Isotherm Analysis

All data analysis and binding isotherm fitting was carried out with OriginPro 2017. All binding constants are rounded to three significant figures and were obtained by fitting of the sensing isotherms to the Langmuir model (Equation S1).

$$\theta = \frac{\left(K_{app} * \left[Cation^{+}\right]\right)}{1 + \left(K_{app} * \left[Cation^{+}\right]\right)}$$

Eqn. S1

In this work, a classical Langmuir model was fitted to all of the obtained cation binding isotherms to enable comparisons of the sensor performance with each cation. A key assumption that underpins the Langmuir model is that binding occurs at discrete, independent binding sites. The model itself cannot and does not distinguish between 1:1 and 2:1 host-guest binding stoichiometry providing that only one binding mode occurs. I.e., excellent fits are expected when 1:1 host-guest binding events occur 100% of the time or equally when 2:1 host-guest binding events occur. A Langmuir-Freundlich model (an adapted form of the Langmuir model, that accounts for surface heterogeneity by describing the distribution of adsorption energy onto heterogeneous surfaces with a parameter, n, that lies between 0-1) was also fitted to all of the binding isotherms to try to account for the complex behaviour observed with the larger alkali metals, but no clear trends in n were observed (see ESI, Table S5). Due to the complexity of these phenomena, the classical Langmuir model was used in all cases to at least facilitate semi-quantitative comparisons between the sensing performance of $B15C5_{SAM}$ for all of the alkali metal cations.

Calibration curves were fitted to a Langmuir-Freundlich model (Equation S2). n represents an inhomogeneity factor here, and it should be noted that when n = 1, Eqn. S2 simplifies to the standard Langmuir model.

$$\theta = \frac{\left(K_{app} * \left[Cation^{+}\right]\right)^{n}}{1 + \left(K_{app} * \left[Cation^{+}\right]\right)^{n}}$$

Eqn. S2

Relative responses (in %) were calculated according to $C_{rel} = (C_A — C_0)/C_0 \times 100.$

S1.8: Limit of Detection (LOD) and Sensitivity Determination

LODs were calculated according to a standard equation given by Eqn. S3 where σ is the standard deviation of the baseline/blank and S is the slope of the linear region of the sensor

response (herein also referred to as "sensitivity"). For continuous capacitive measurements, σ was determined from the root-mean-square deviation of a linear fit of 10 data points in the initial baseline of the sensograms, immediately preceding the response of the first addition. S was determined from the slope of a linear fit to the linear regime of each respective binding isotherm, with a linear range of 0-1 mM, as representatively shown in Figure S1.

$$LOD = \frac{3\sigma}{S} \quad \text{Eqn. S3}$$

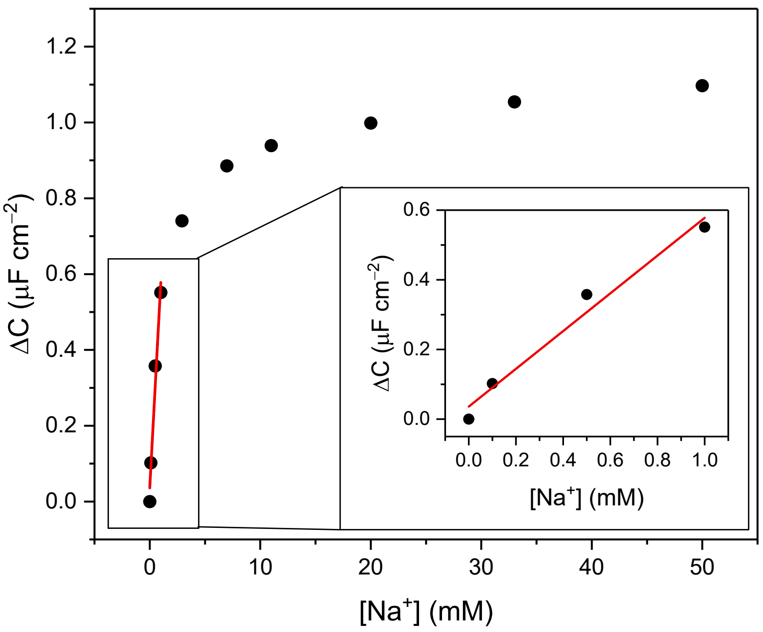

Figure S1. Representative binding isotherm for **B15C5$_{SAM}$** in response to increasing concentrations of Na^+ (up to 50 mM), with a linear fit to the linear regime of the binding isotherm (0-1 mM) which was used to calculate the limit of detection according to Equation S3.

S1.9: Preparation of Real-world Water Samples

Tap water sample was taken directly from the tap in the laboratory where this work was conducted on 06/04/2023 (Thames Water). The freshwater sample was collected by Dr. Robert Barber from the Blue Lough in the Mourne Mountains on 07/04/2023. Both samples were filtered with 0.45 μM syringe filters prior to measurement to remove any particulates which may affect the sensing performance, but it should be noted that this will not, of course, remove any of the ionic species expected to interfere with ion binding (Li^+, K^+ etc.). The samples were then spiked with 100 mM TEACl, and aliquots of increasing Na^+ concentration were prepared for continuous flow ion sensing studies.

S2: Synthesis and Characterisation of B15C5

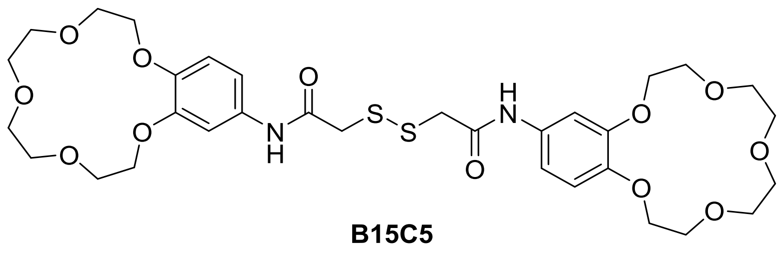

B15C5

Synthetic procedure: Dithioglycolic acid (32 mg, 0.177 mmol, 1 equiv.), 4'-aminobenzo-15-crown-5 (100 mg, 0.354 mmol, 2 equiv.), 4-dimethylaminopyridine (59 mg, 0.531 mmol, 3 equiv.) and 1-ethyl-3-(3-dimethylaminopropyl)carbodiimide hydrochloride (0.425 mmol, 2.4 equiv.) were dissolved in 5 mL of anhydrous DCM and reacted at room temperature overnight. Subsequently, 25 mL DCM were added and the organic phase was washed 3 times with 5% aqueous HCl and then once with H_2O. Following drying over $MgSO_4$ and removal of the solvent in vacuo, the product was purified by alumina column chromatography (DCM to 3.5% MeOH in DCM gradient). The product was obtained as a yellow solid (83 mg, 0.116 mmol, 66%).

^{1}H NMR (600 MHz, $CDCl_3$/MeOD 2:1 (v/v)): δ 7.30 (d, *J* = 2.4 Hz, 2H), 7.05 (dd, *J* = 8.6, 2.4 Hz, 2H), 6.82 (d, *J* = 8.6 Hz, 2H), 4.08 (ddd, *J* = 8.8, 5.1, 3.3 Hz, 8H), 3.86 (tt, *J* = 6.7, 3.6 Hz, 8H), 3.75 – 3.68 (m, 16H), 3.59 (s, 4H).

^{13}C NMR (151 MHz, $CDCl_3$/MeOD 2:1 (v/v)) δ 167.81, 149.10, 145.86, 132.23, 114.65, 112.84, 107.09, 70.71, 70.65, 70.31, 69.52, 69.40, 69.34, 68.68, 43.56.

HRMS (ESI+ve): m/z: 735.2221 ($[M+Na]^+$, $C_{32}H_{44}O_{12}N_2NaS_2$ requires 735.2228).

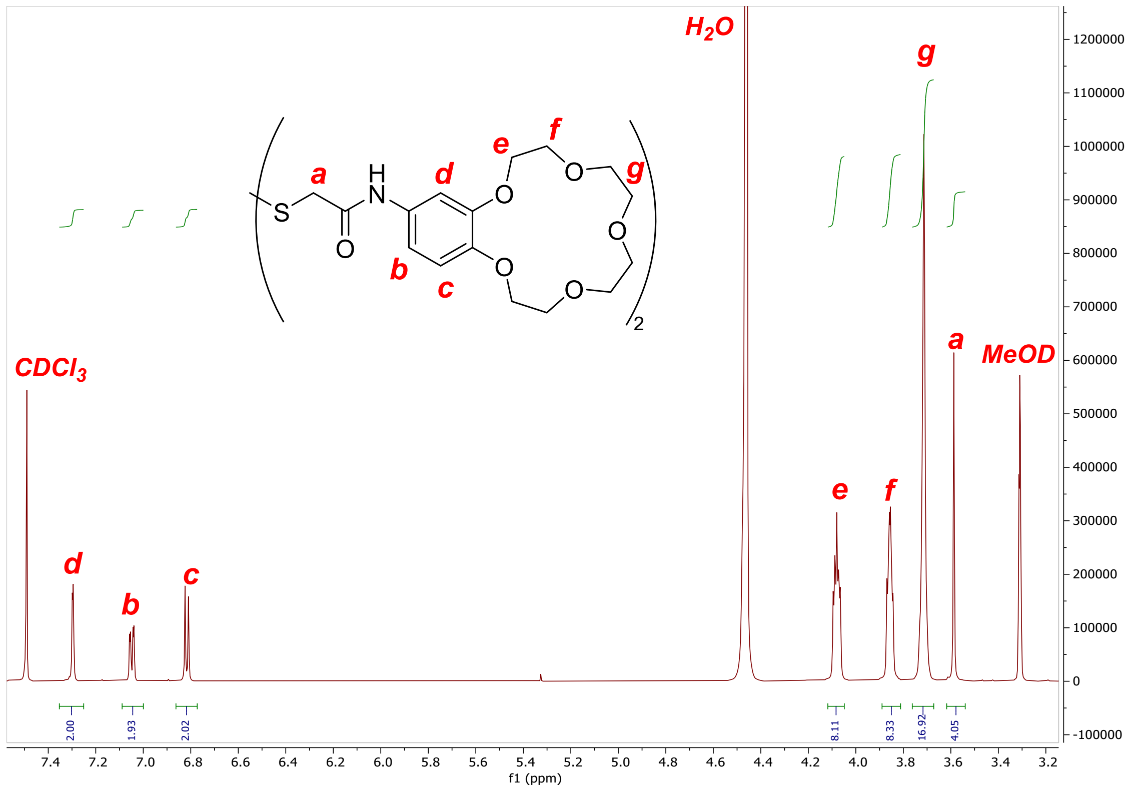

Figure S2. ^{1}H-NMR of **B15C5** in $CDCl_3$/MeOD 2:1 (v/v).

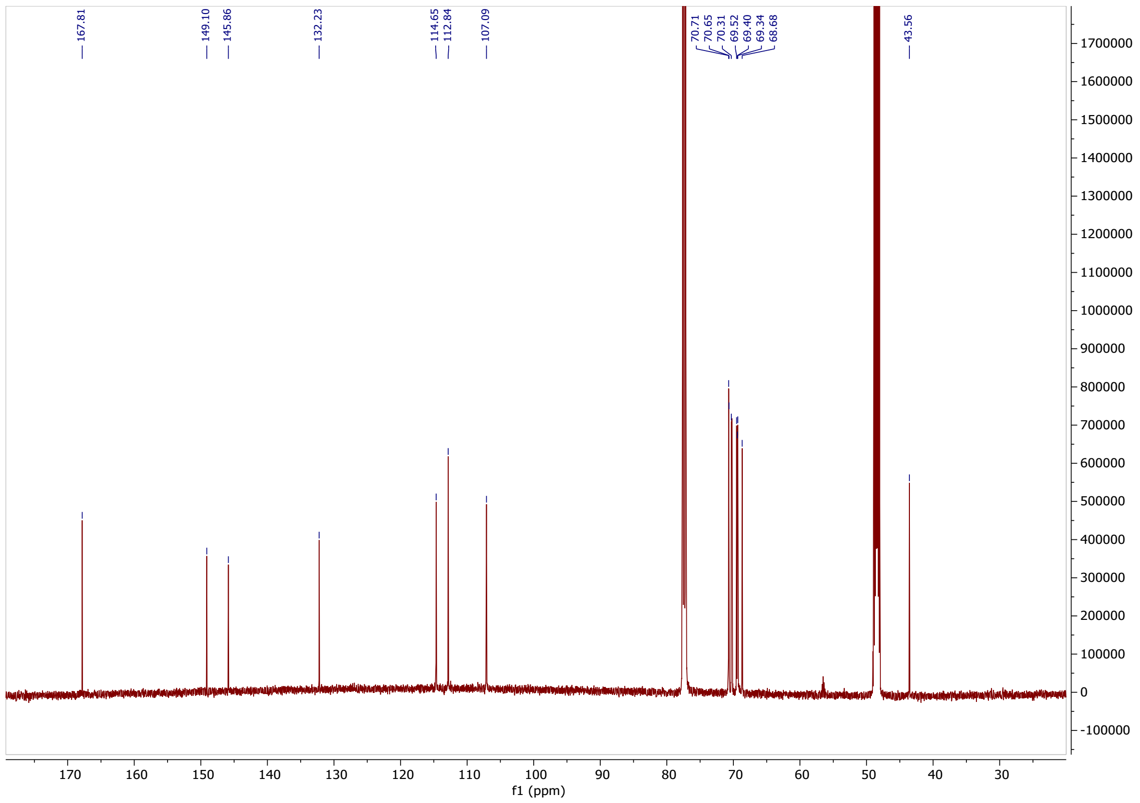

Figure S3. ^{13}C-NMR of **B15C5** in $CDCl_3$/MeOD 2:1 (v/v).

S3: Film Formation and Characterisation of B15C5$_{SAM}$

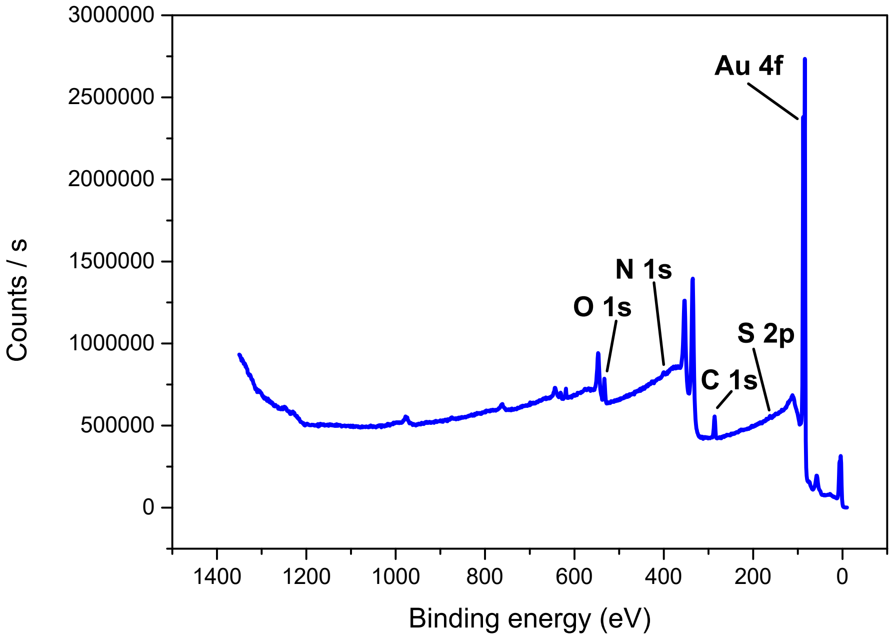

Figure S4. Low resolution survey XPS spectrum of **B15C5$_{SAM}$**.

Table S1. Experimental and theoretical elemental composition of **B15C5$_{SAM}$** determined by XPS. Values were normalized to O = 6. Errors represent one standard deviation of triplicate measurements.

Element	Experimental	Theoretical
**C**	17.18 ± 0.80	16
**O**	6 ± 0.21	6
**N**	1.71 ± 0.83	1
**S**	0.55 ± 0.13	1

The slightly poorer agreement observed for N and S presumably arises from an overall low abundance and low sensitivity at these low concentrations, as well as attenuated sensitivity due to their relatively “deeper” location within the film and penetration attenuation of the X-rays. The individual peak fits for C and O are generally good (see Table S2). The S binding energies are consistent with chemisorption of the disulfide groups to the Au electrode surface.

Table S2. Peak binding energies and experimental and theoretical ratios for different chemical environments of C, O, N and S of **B15C5$_{SAM}$**. For S only one environment is observed giving rise to two peaks with an area ratio of 2:1 due to spin orbit coupling.

Element		peak BE (eV)	Exp.	Theor.
**C**	*C*=C	284.52 ± 0.03	1.72 ± 0.268	3
	C-O, *C*-N, *C*-S	286.18 ± 0.03	8.07 ± 2.24	12
	C=O	288.81 ± 0.39	1	1
**O**	C=*O*	531.18 ± 0.07	1	1
	C-*O*	532.94 ± 0.01	5.55 ± 0.77	5
**N**	*N*-C=O	400.03 ± 0.16	/	/
**S**	*S*-C	162.25 ± 0.07	/	/
		163.55 ± 0.11	/	/

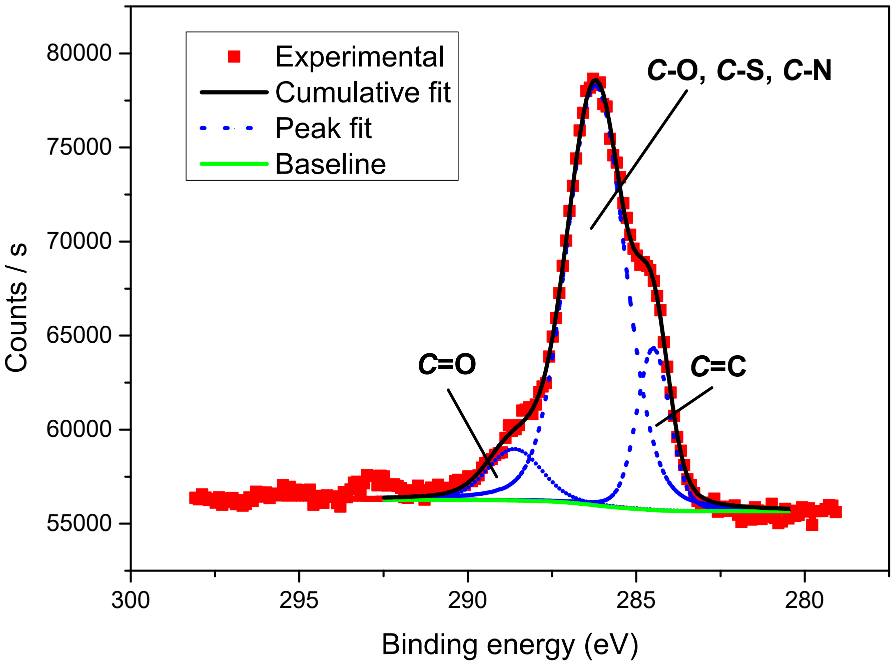

Figure S5. High resolution C1s XPS spectrum of **B15C5$_{SAM}$** including peak fits.

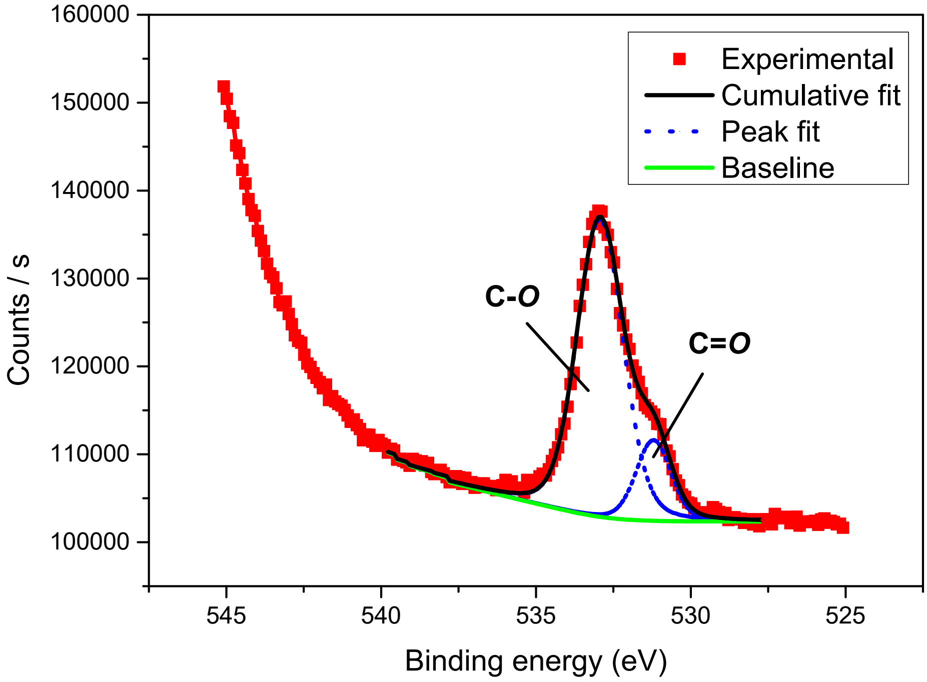

Figure S6. High resolution O1s XPS spectrum of **B15C5**$_{\mathbf{SAM}}$ including peak fits.

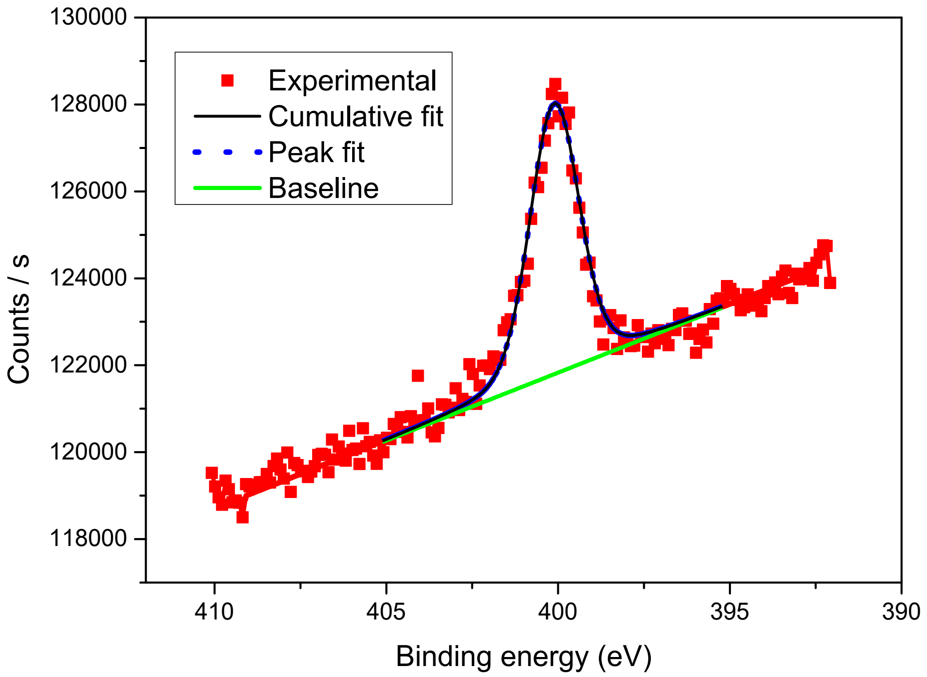

Figure S7. High resolution N1s XPS spectrum of **B15C5**$_{\mathbf{SAM}}$ including peak fits.

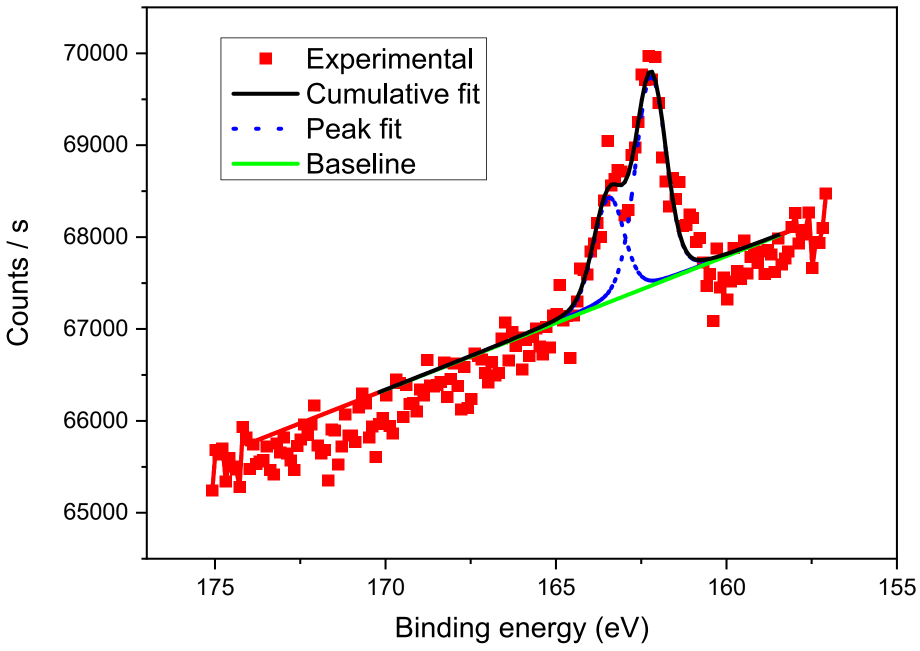

Figure S8. High resolution S2p XPS spectrum of **B15C5$_{SAM}$** including peak fits.

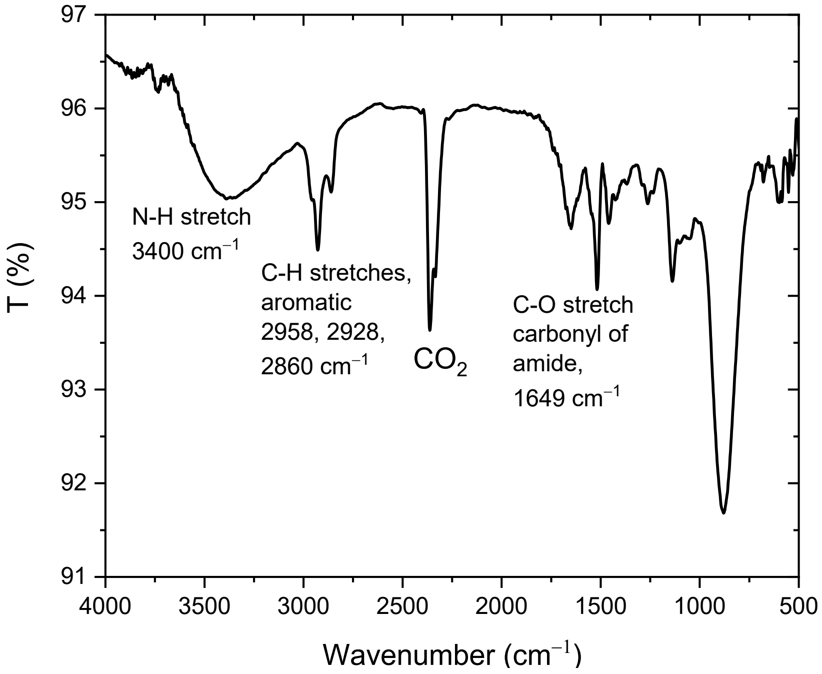

Figure S9. ATR-FTIR spectrum of **B15C5$_{SAM}$** on Au.

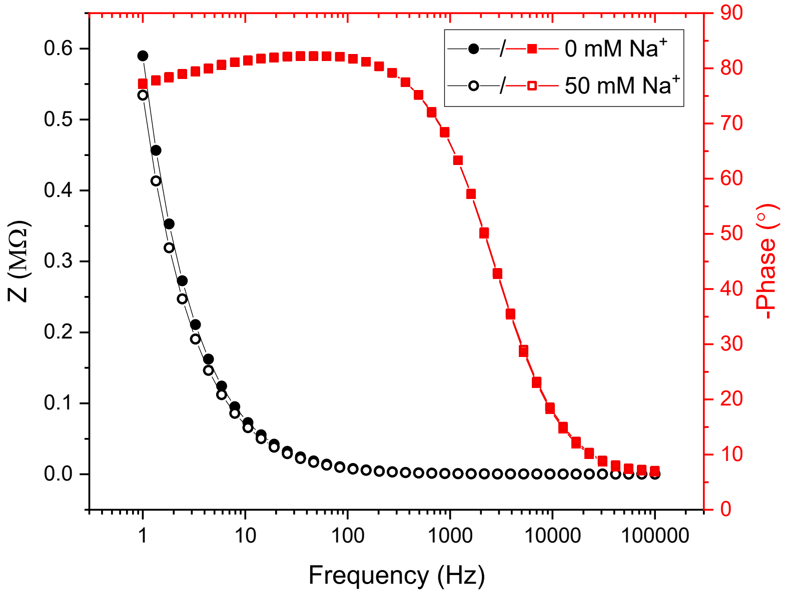

Figure S10. Bode plots of impedance (black) and phase (red) vs. frequency of **B15C5$_{SAM}$** in 100 mM TEACl in H_2O the presence (hollow) and absence (solid) of 50 mM Na^+.

S4 Capacitive Characteristics of Receptive Interfaces

Capacitive processes are probed at a fixed DC potential (herein the open-circuit potential, OCP), upon which a small amplitude AC perturbation (10 mV) across a range of frequencies (1-100,000 Hz) is superimposed. The non-Faradaic characteristics and resolved film capacitance, *C* of a receptive interface (e.g. a self-assembled monolayer on gold, as used herein) can most simply be described by a plate capacitor Helmholtz model (Equation 1, where ε_r is film dielectric, A is electrode surface area, and d is receptive film thickness). As ε_0, d and A are expected to remain constant upon ion binding, assuming there are no significant film rearrangements induced by specific target recruitments that could affect d, *C* is therefore directly proportional to the dielectric constant of the film, ε_r. Predictably, then, ion recruitment to the receptive molecular film such as Na^+ binding to B15C5 (and any associated enhanced hydration due to introduction of charged species)[6] increases the film dielectric constant, and thus induces an increase in the value of *C* (see Figure 1B).[6, 9, 10]

Further light can be shed on this model, as the native *C* of the film in the absence of target ions can be separated into its constituent elements, namely C_m (which is itself comprised of two constituent capacitors in series: C_b and C_{dl}) and C_t, which are schematised in Figure S12. Here, C_m is the native capacitance of the film itself, with contributions from C_b, the dielectric capacitance of the film in the absence of any ions, and the double layer capacitance, C_{dl}, which accounts for the interactions between the electrolyte and conductive surface (i.e., the gold electrode surface) in this model. Note that a sufficiently short linker group was chosen in the design of **B15C5** to preclude contributions from a so-called linker capacitor ($C_{b,linker}$) in series, which is invariable to ion binding at the SAM and has been shown to dampen specific response magnitudes.[11] C_t describes the capacitance of *non-specific* ion ingress (i.e., the supporting electrolyte, TEACl) into the film.[12, 13] Each of these elements are associated with spectrally-resolved frequency domains, as highlighted in the capacitive Nyquist plots in Figure S12B and Bode plots in Figure S12C. Namely, C_m is resolved at high frequencies (> 10,000 Hz), and C_t at intermediate frequencies (typically 50-200 Hz), as represented by the semi-circular region of the capacitive Nyquist plot in Figure S12B (black trace).

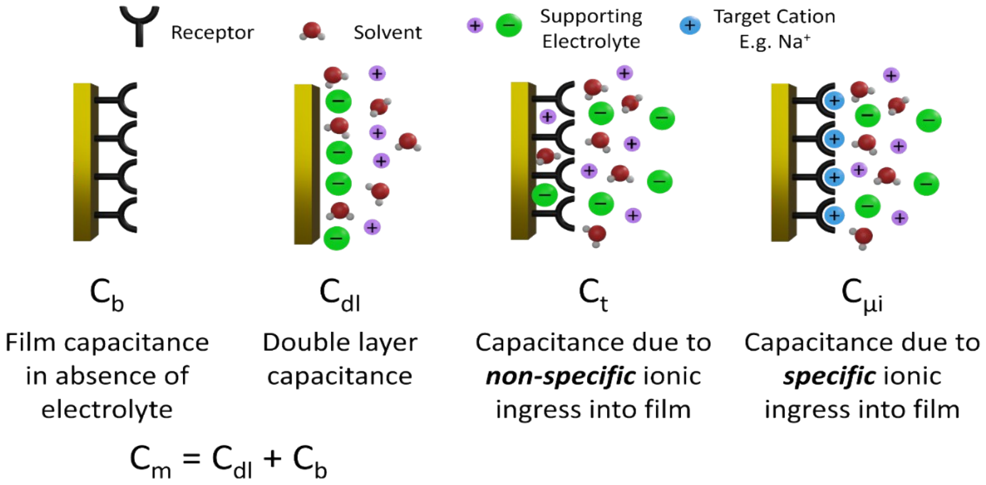

Figure S11. Schematic depiction of receptive film capacitors in the presence and absence of a specific target.

Upon addition of target ion (herein Na^+, or other alkali metals), a so-called ion binding capacitance, denoted $C_{\mu i}$, is additionally introduced which represents *specific* ionic ingress into the film, which can be considered a unique case of, or contributor to, C_t upon ion binding. As such, C_t and $C_{\mu i}$ are represented as parallel elements, and the interface (both in the absence or presence of target ion) can be described by the equivalent circuit $R_s(C_m[R_tC_t])$, as proposed previously.[6,9] Importantly, it should be noted that C_m has an essentially negligible contribution to *C* when densely-packed molecular films are utilised, or is at the very least unchanged when target ions are added at constant ionic strength (shown by the minimal change in *C* at the high frequency domain between the black and blue traces in Figure S12B/C). The film capacitance, *C* can thus be obtained as the real component of the capacitance at the turning point of the hemispherical region of capacitive Nyquist plots, at the corresponding intermediate frequency denoted f_c (i.e., the frequency corresponding to $C_t/C_{\mu i}$, typically 50-150 Hz. The imaginary component is also sensitive to ion recruitment (SI Figure S12B) and could equally be used as a sensory readout. However, specific ion binding-induced changes are generally greater for the real component, thus the real component of *C* was chosen as the primary sensory readout to ensure the highest signal-to-noise-ratio for these measurements. The baseline film capacitance of **B15C5$_{SAM}$** in the absence of target cations and under standard, static conditions was evaluated to be 3.68 ± 0.18 μF cm^{-2}, in good agreement with other densely-packed, receptive SAMs.[11] Expectedly, *C* increases upon target ion recruitment in accordance with Equation 1, as representatively shown in Figure S12B by the shift in the inflection point between the capacitive Nyquist plots of **B15C5$_{SAM}$** in the absence (black trace) and presence (blue trace) of 50 mM Na^+. As detailed above, changes in *C* are thus expected

to predominantly result from changes in $C_t/C_{\mu i}$ (non-specific or specific ionic ingress into the molecular film, respectively).

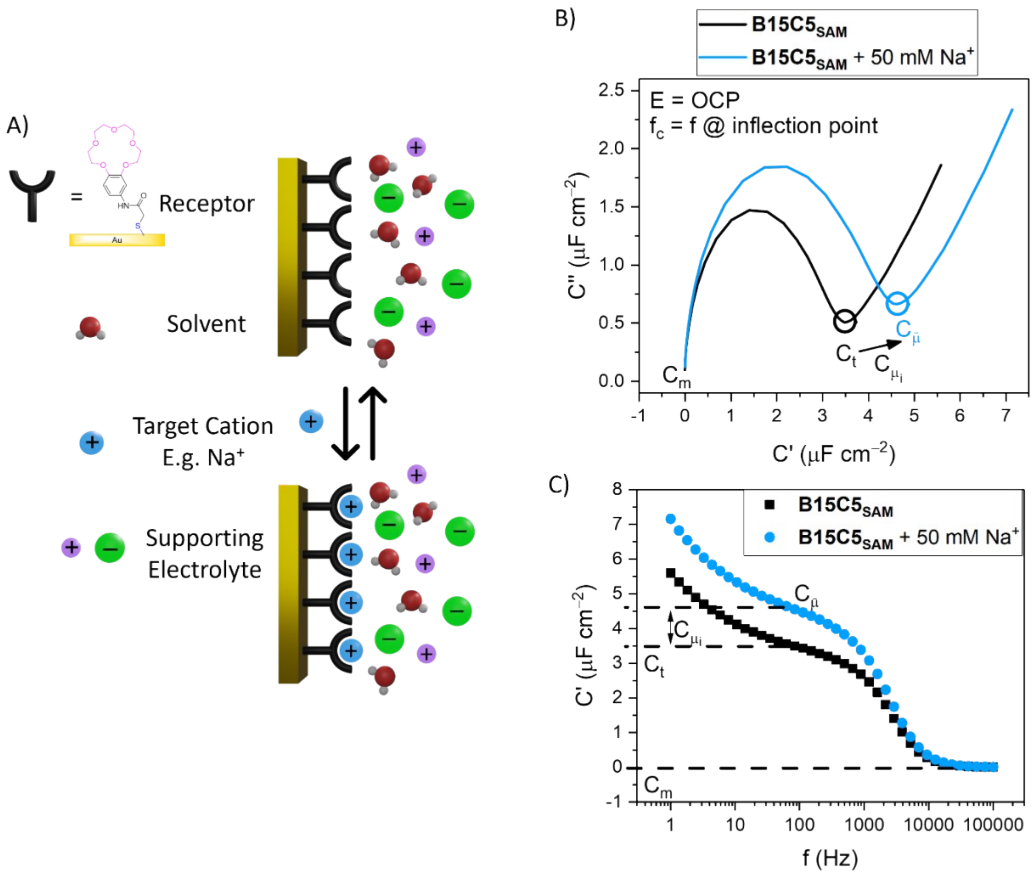

Figure S12. A) Schematic depiction of **B15C5$_{SAM}$** in the presence and absence of a specific target and B) corresponding capacitive Nyquist and C) Bode plots. Each data point represents a measurement at a different AC frequency (1-100,000 Hz). The film capacitance is then obtained as the real capacitance C′ at the frequency of the inflection point (f_c) of the capacitive Nyquist plot, approximately the semicircle diameter.

S5 Static Cation Sensing Studies

The cation sensing performance of the **$B15C5_{SAM}$** molecular films was first assessed under static conditions whereby a 3-electrode cell (Au disc working, Pt counter and Ag|AgCl pseudo reference electrode) was assembled, with 100 mM TEACl in water as supporting electrolyte. All responses were determined from the shift in the real component (C') of the inflection point in the capacitive Nyquist plots (see Figure S13) and are stated relative to the initial C' in the absence of the target cation ([M^+] = 0 mM). The progressive increase of C' upon addition of increasing concentrations of M^+ corresponds to specific recruitment of cationic species into the **$B15C5_{SAM}$** interface (Figure S13), which expectedly increases the associated dielectric constant of the molecular films (which are related according to Equation 1).[11]

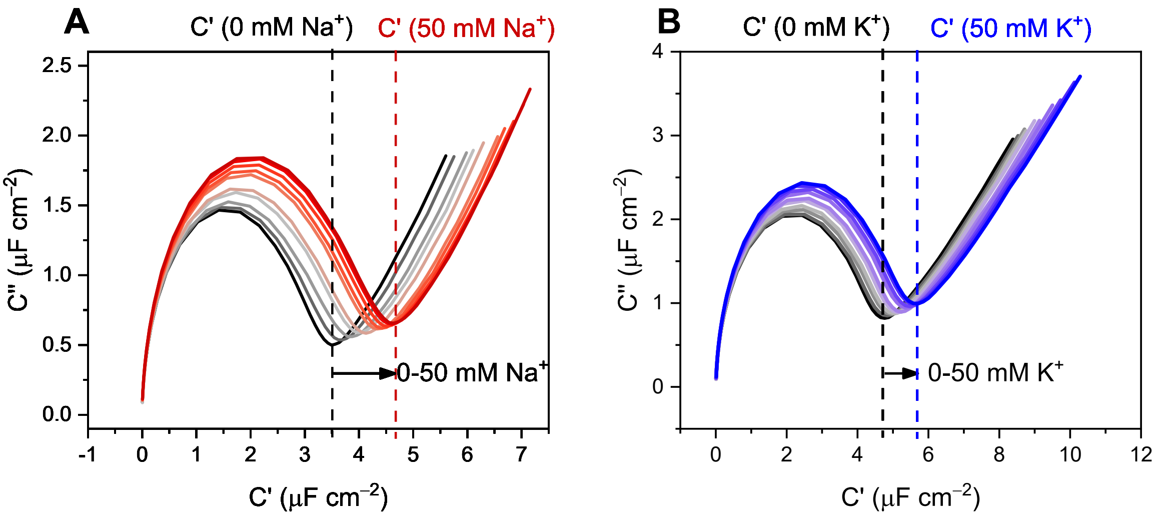

Figure S13. Capacitive Nyquist plots to display the shift in C' at **$B15C5_{SAM}$** receptive films induced by addition of increasing concentrations of A) Na^+ and B) K^+ in 100 mM TEACl in H_2O, under static conditions.

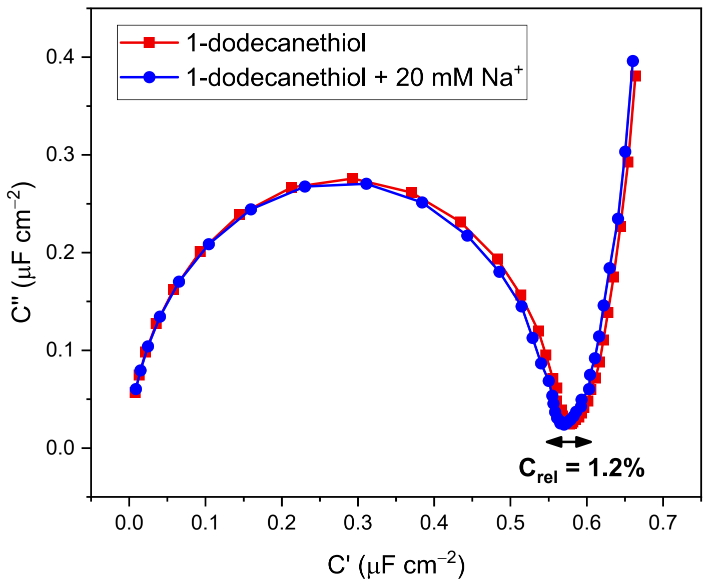

Figure S14. Capacitive Nyquist plots of **1-dodecanethiol$_{\mathbf{SAM}}$** in 100 mM TEACl in H_2O in the presence (blue circles) and absence (red squares) of 20 mM NaCl, highlighting the negligible change in *C* at a model non-receptive film in the presence of a significant target cation concentration.

Table S3. Binding constants of **B15C5$_{\mathbf{SAM}}$** from Langmuir fitting (Equation S1) of response isotherms with increasing concentrations of Na^+ and K^+ under static conditions. Errors represent one standard deviation of at least three independent repeats. The interface sensitivity was obtained by analysis of the pseudolinear region at low concentration (0–1 mM [M^+]), which was then used to calculate the limit of detection (LOD). Further details on these calculations are included in the Supporting Information, Section S1.8.

	ΔC_{max} (μF cm^{-2})	K_{Static} (M^{-1})	Sensitivity (μF cm^{-2} mM^{-1})	LOD (μM)
**Na^+**	1.66 ± 0.25	434 ± 205	0.44 ± 0.06	12.3 ± 3.6
**K^+**	0.61 ± 0.06	380 ± 188	0.23 ± 0.04	11.8 ± 3.1

S6 Microfluidic Flow Setup

Continuous flow measurements were performed in an analogous manner to previous reports with the same syringe pump (Harvard Apparatus PHD 2000), injector (Rheodyne® Model 9725), and tubing (Cole-Parmer PEEK tubing or Darwin Microfluidics PTFE Tubing, 1.5875 mm outer diameter, 0.5 mm inner diameter).[14, 15]

In previous reports, a 3-electrode electrochemical cell comprised of a Pt counter, Au disc working and Ag|AgNO3 fritted reference electrode was utilised for all static and continuous flow anion sensing studies.[14, 15] In this work, however, notable inconsistencies were observed with this setup, with both the baseline *C* (in the absence of target cations), and the *C* response profiles and magnitudes unexpectedly displaying a significant and reproducible flow-rate dependence. Therefore, an adapted cell design was used to house and employ a Ag|AgCl wire pseudoreference electrode instead of a fritted reference electrode (see Figure S15). When this setup was utilised, the flow rate dependence of both the baseline C and cation-induced C responses of **B15C5**$_{\mathbf{SAM}}$ were eliminated.

Flow cell designs were generated with Autodesk Fusion 360 (see Figure S15 for annotated blueprint of design), which were then rendered into compatible (printable) designs in CHITUBOX. Flow cells were produced with an Elegoo Mars 3D printer using ANYCUBIC 3D Printing UV Sensitive Resin Basic (Clear). A Pt wire (counter electrode) and a Ag|AgCl pseudoreference wire electrode were held in place in a channel adjacent to the main chamber with epoxy resin (Araldite Rapid), and the Au disc electrode functionalised with **B15C5**$_{\mathbf{SAM}}$ was inserted before each experiment, giving an airtight cell with an approximate inner chamber volume of 10 µL (Figure S15).

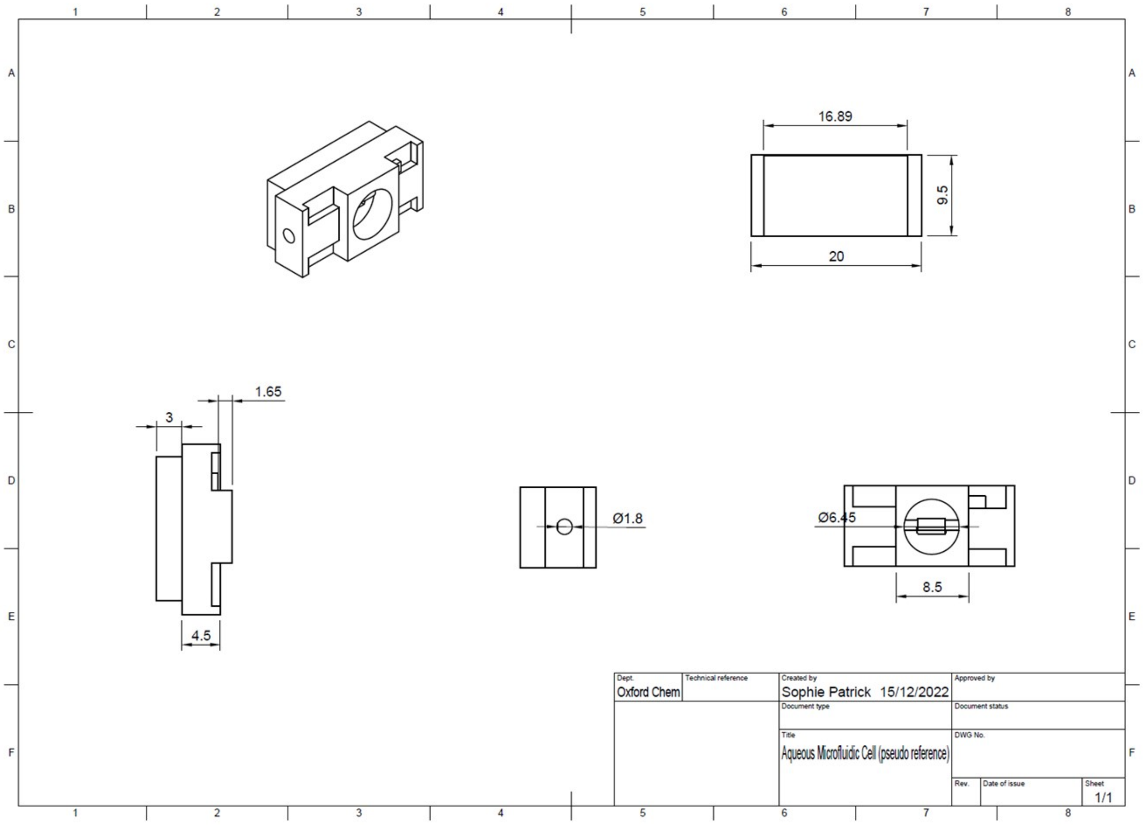

Figure S15. Blueprint schematic of new flow cell design.

S7 Real-time Ion Sensing Under Continuous Flow

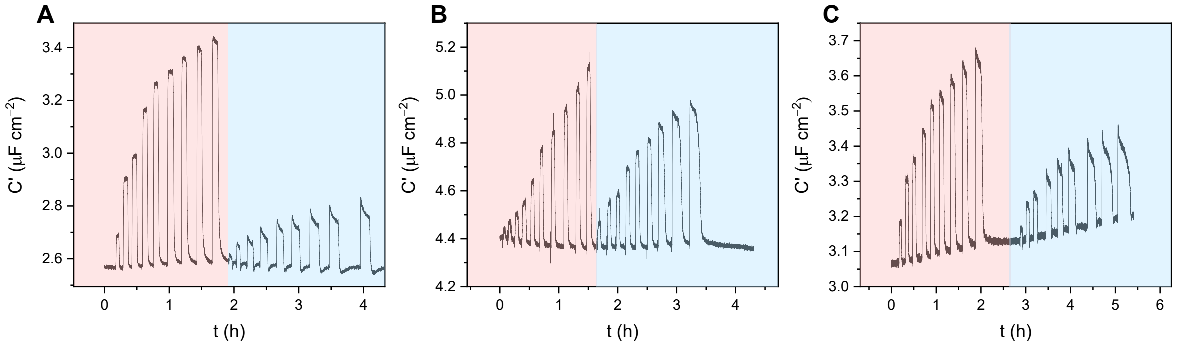

Figure S16. Representative sensograms of the response of **B15C5$_{SAM}$** in 100 mM TEACl in H_2O to increasing concentrations (in order: 0.1, 0.5, 1, 3, 7, 11, 20, 33 and 50 mM MCl) of A) Na^+ (left, red) and K^+ (right, blue), B) Li^+ (left, red) and Rb^+ (right, blue) and C) NH_4^+ (left, red) and Cs^+ (right, blue).

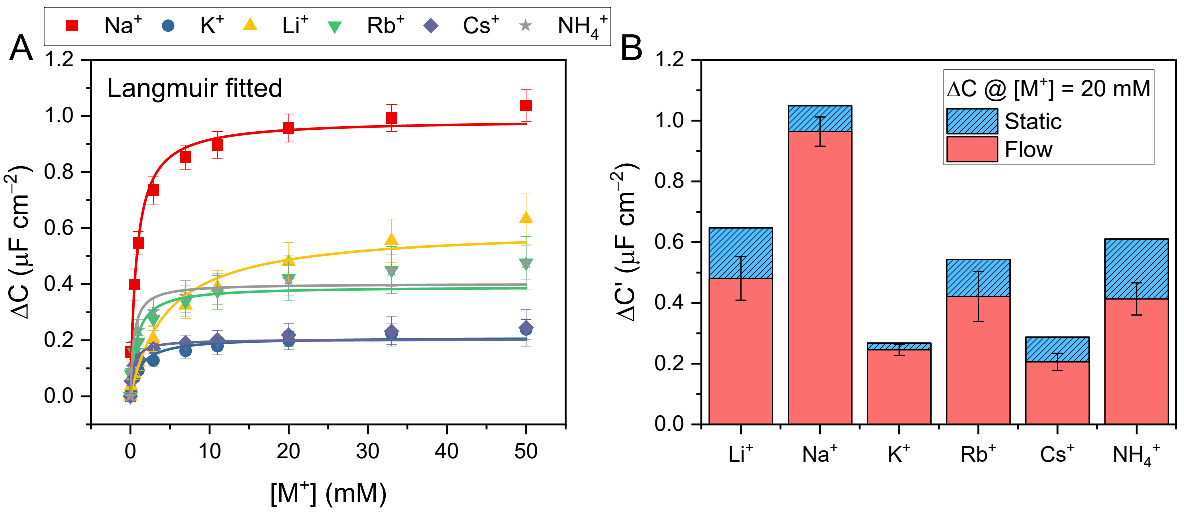

Figure S17. Additional data for Figure 4. Response isotherms determined from the shift in C' in response to increasing concentrations of Na^+ (red squares), K^+ (blue circles), Li^+ (yellow triangles), Rb^+ (green inverted triangles), Cs^+ (purple diamonds) and NH_4^+ (grey stars) under continuous flow at 100 μL min^{-1}. Isotherms were fitted to a Langmuir model (see SI Section S1.6 for further details, and Equation S1). Errors represent one standard deviation of three independent repeats. B) Selectivity studies with **B15C5$_{SAM}$** under continuous flow (red) and static (blue) conditions upon addition of 20 mM of Li^+, Na^+, K^+, Rb^+ and Cs^+ (in order of increasing ionic diameter ranging from 1.44-3.34 Å, cf. 15C5 cavity diameter = 1.84 Å).[2]

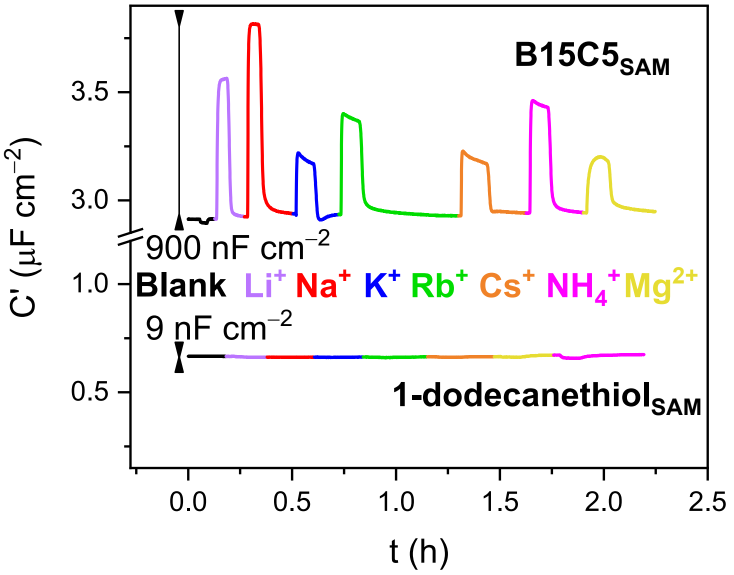

Figure S18. Comparison of the responses determined at receptive **B15C5$_{SAM}$** films and a non-receptive control interface, **1-docanethiol$_{SAM}$** upon addition of a blank sample (100 mM TEACl), and 20 mM Li^+, Na^+, K^+, Rb^+, Cs^+, NH_4^+ and Mg^{2+}, added as MCl salts. As shown, the control interface exhibits negligible responses (< 9 nF, C_{rel} < 0.2%) to any of the cations investigated.

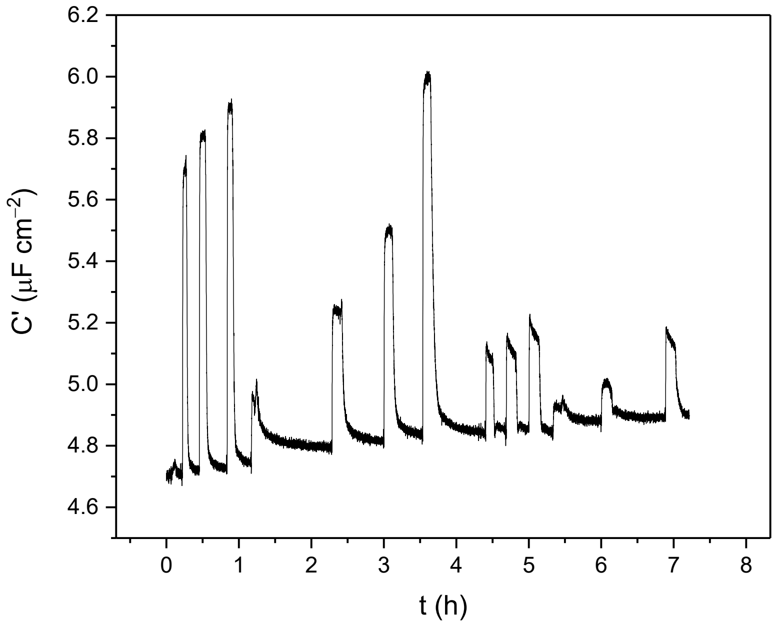

Figure S19. Sensogram displaying the response of **B15C5$_{SAM}$** to: 5 mM Na^+, 10 mM Na^+, 20 mM Na^+, 10 mM [2.2.2]-cryptand, 5 mM Na^+ + 10 mM [2.2.2]-cryptand, 10 mM Na^+ + 10 mM [2.2.2]-cryptand, 20 mM Na^+ 10 mM [2.2.2]-cryptand. Then 5 mM K^+, 10 mM K^+, 20 mM K^+, 5 mM K^+ + 10 mM [2.2.2]-cryptand, 10 mM K^+ + 10 mM [2.2.2]-cryptand, 20 mM K^+ 10 mM [2.2.2]-cryptand.

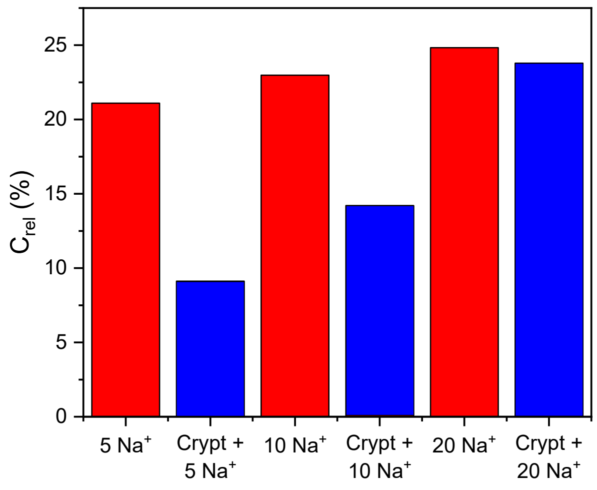

Figure S20. Competition studies of **B15C5$_{SAM}$** with [2.2.2]-cryptand for Na^+. The red bars represent the responses to Na^+ aliqouts (5, 10 and 20 mM) alone, and the blue bars denote responses to Na^+ (5, 10 and 20 mM) aliquots which were precomplexed with 10 mM [2.2.2]-cryptand.

Table S4. Binding constants of **B15C5$_{SAM}$** from Langmuir fitting (Equation S1) of response isotherms with increasing concentrations of a range of alkali metals (Li^+, Na^+, K^+, Rb^+, Cs^+ and NH_4^+) under continuous flow at 100 µL min^{-1}. Errors represent one standard deviation of at least three independent repeats. The interface sensitivity was obtained by analysis of the pseudolinear region at low concentration (0–1 mM [M^+]), which was then used to calculate the limit of detection (LOD).

	ΔC_{max} (µF cm^{-2})	K (M^{-1})	Sensitivity (µF cm^{-2} mM^{-1})	LOD (µM)
Li^+	0.63 ± 0.09	133 ± 12	0.11 ± 0.02	14.7 ± 2.7
Na^+	1.03 ± 0.06	1252 ± 341	0.52 ± 0.05	4.0 ± 1.2
K^+	0.29 ± 0.02	555 ± 58	0.09 ± 0.01	27.4 ± 4.7
Rb^+	0.48 ± 0.09	763 ± 197	0.18 ± 0.02	9.3 ± 0.8
Cs^+	0.24 ± 0.07	1929 ± 640	0.12 ± 0.01	18.2 ± 7.5
NH_4^+	0.47 ± 0.06	1232 ± 357	0.21 ± 0.04	11.0 ± 0.8

Table S5. Binding constants of **B15C5$_{SAM}$** from Langmuir-Freundlich fitting (Equation S2) of response isotherms with increasing concentrations of a range of alkali metals (Li^+, Na^+, K^+, Rb^+, Cs^+ and NH_4^+) under continuous flow at 100 μL min^{-1}. Errors represent one standard deviation of at least three independent repeats. The interface sensitivity was obtained by analysis of the pseudolinear region at low concentration (0–1 mM [M^+]), which was then used to calculate the limit of detection (LOD).

	ΔC_{max} (μF cm^{-2})	K (M^{-1})	n	Sensitivity (μF cm^{-2} mM^{-1})	LOD (μM)
**Na^+ (static)**	1.67 ± 0.25	72 ± 42	0.61 ± 0.04	0.44 ± 0.06	12.3 ± 3.6
**K^+ (static)**	0.61 ± 0.06	248 ± 164	0.66 ± 0.07	0.23 ± 0.04	11.8 ± 3.1
**Li^+**	0.63 ± 0.09	39 ± 16	0.63 ± 0.07	0.11 ± 0.02	14.7 ± 2.7
**Na^+**	1.03 ± 0.06	1012 ± 382	0.72 ± 0.07	0.52 ± 0.05	4.0 ± 1.2
**K^+**	0.29 ± 0.02	210 ± 34	0.57 ± 0.01	0.09 ± 0.01	27.4 ± 4.7
**Rb^+**	0.48 ± 0.09	262 ± 151	0.50 ± 0.03	0.18 ± 0.02	9.3 ± 0.8
**Cs^+**	0.24 ± 0.07	1335 ± 544	0.57 ± 0.09	0.12 ± 0.01	18.2 ± 7.5
**NH_4^+**	0.47 ± 0.06	491 ± 439	0.47 ± 0.07	0.21 ± 0.04	11.0 ± 0.8

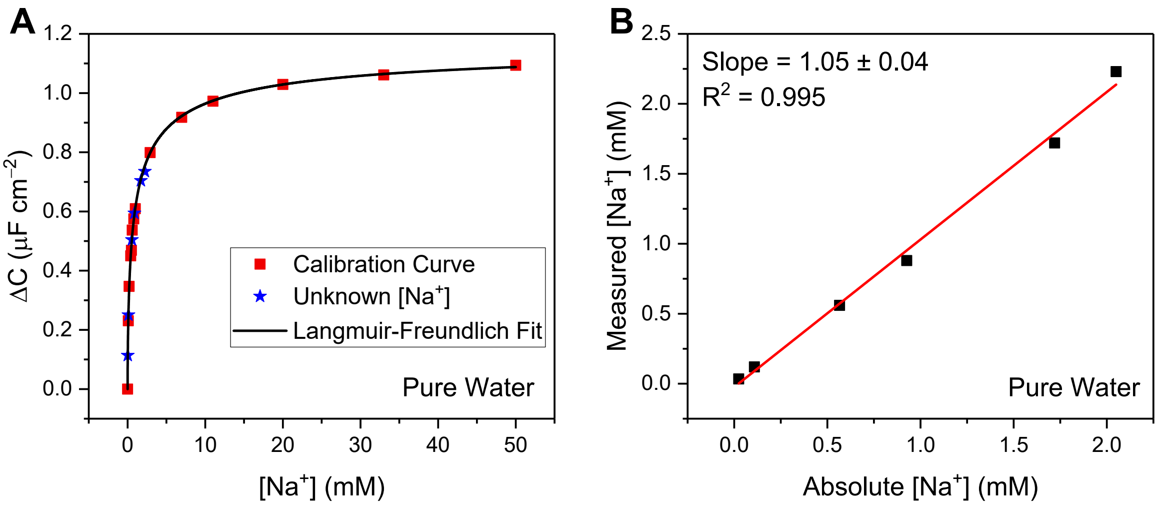

Figure S21. A) Responses to samples of unknown concentration in pure water superimposed onto a calibration curve obtained prior to blind studies, and fitted to a Langmuir-Freundlich model (Equation S2) which was chosen to provide the closest fit to the calibration data set. B) Correlation plot showing the excellent agreement between absolute and measured [Na^+] values for a range of blindly unknown concentrations (0.034, 0.12, 0.56, 0.88, 1.72 and 2.23 mM) in pure water (100 mM TEACl as supporting electrolyte). Measured [Na^+] values were estimated from comparison to the calibration curve shown in Figure S21A.

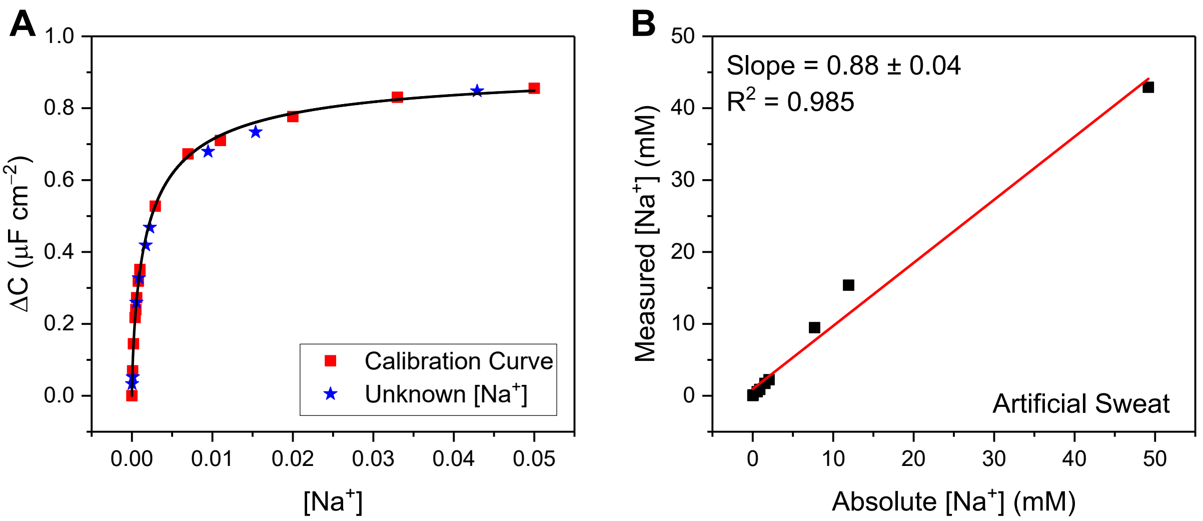

Figure S22. A) Responses to samples of unknown concentration in artificial sweat (100 mM TEACl, 11.3 mM lactic acid, 21.6 mM urea in pure water) superimposed onto a calibration curve obtained prior to blind studies, and fitted to a Langmuir-Freundlich model (Equation S2) which was chosen to provide the closest fit to the calibration data set. B) Correlation plot showing the excellent agreement between absolute and measured [Na^+] values for a range of blindly unknown concentrations (0.034, 0.12, 0.56, 0.88, 1.72, and 2.23 mM, as well as additional concentrations: 9.47, 15.38 and 42.91 mM). Measured [Na^+] values were estimated from comparison to the calibration curve shown in Figure S22A.

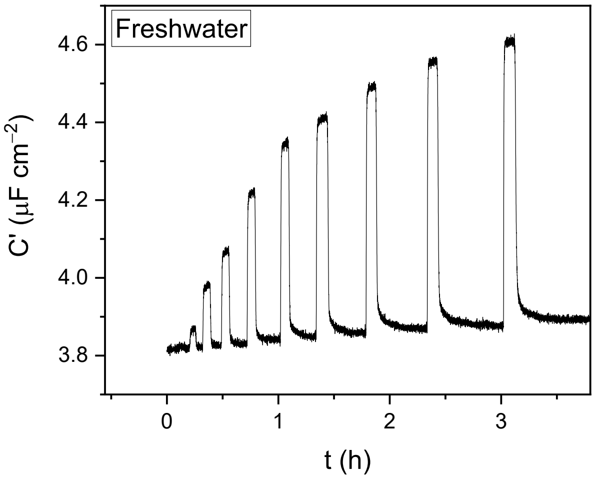

Figure S23. Sensogram displaying the response of **B15C5$_{SAM}$** to increasing concentrations of Na^+ up to 50 mM in fresh water (with 100 mM TEACl as supporting electrolyte).

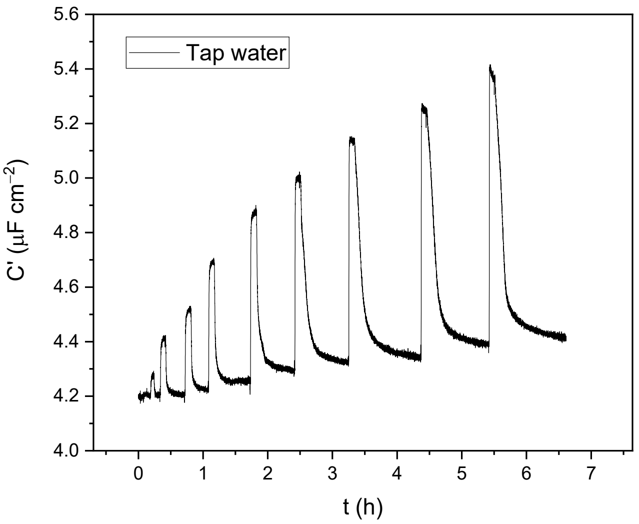

Figure S24. Sensogram displaying the response of $\mathbf{B15C5_{SAM}}$ to increasing concentrations of Na^+ up to 50 mM in tap water (with 100 mM TEACl as supporting electrolyte).

The slight attenuation in response magnitude (and resolved Na^+ binding constant) with respect to studies in deionised water were attributed to some degree of interference from other cations present in solution (presumably K^+, in particular, or Ca^{2+} or Mg^{2+}).[16]

S8 Resolving Different Binding Modes

At a flow rate of 100 µL min^{-1} and a cell volume of ≈ 10 µL, any mixing of the supporting electrolyte and analyte solution within the cell is expected to conclude within 2 s of the analyte solvent front reaching the cell. Therefore, given the high temporal resolution, the data used for this analysis of kinetics predominantly reflects binding and not any artefacts of mixing.

To approximate cation binding and dissociation rate constants, the first 10 consecutive data points of each response > 0.03 uF cm^{-2} from the baseline *C* were fitted to a linear model. 0.03 uF cm^{-2} here correlates to the smallest change observed (0.1 mM K^+), *i.e.*, the smallest change which can be confidently attributed to a significant and specific capacitance response due to cation binding.

Table S6. Slopes determined from linear fits to the first 10 data points of the association regime of binding/recovery with 1, 3 and 50 mM of Li^+, Na^+, K^+, Rb^+, Cs^+ and NH_4^+ (corresponding to the linear fits shown in Figures S25-S30A, C, E, G and I).

	Initial Response Slope (nF cm^{-2} s^{-1})					
**[M$^+$] (mM)**	**Li^+**	**Na^+**	**K^+**	**Rb^+**	**Cs^+**	**NH_4^+**
**0.1**	1.3	3.9	N/A	2.6	N/A	4.2
**1**	4.2	21.9	3.5	8.9	6.4	12.8
**3**	8.0	34.7	6.6	15.2	9.7	18.6
**11**	16.7	52.2	9.0	24.1	10.5	28.2
**50**	37.2	66.0	14.9	34.0	12.9	32.5

Table S7. Slopes determined from linear fits to the first 10 data points of the dissociation regime of binding/recovery with 1, 3 and 50 mM of Li^+, Na^+, K^+, Rb^+, Cs^+ and NH_4^+ (corresponding to the linear fits shown in Figures S25-S30B, D, F, H and J).

	Initial Washing Slope (nF cm^{-2} s^{-1})					
**[M$^+$] (mM)**	**Li^+**	**Na^+**	**K^+**	**Rb^+**	**Cs^+**	**NH_4^+**
**0.1**	1.1	3.4	1.1	2.2	N/A	3.2
**1**	3.6	9.0	2.7	5.3	3.1	5.5
**3**	4.1	9.2	2.5	5.4	2.9	4.9
**11**	6.8	8.3	2.4	4.0	0.2	3.5
**50**	6.7	4.8	2.6	3.8	0.2	0.7

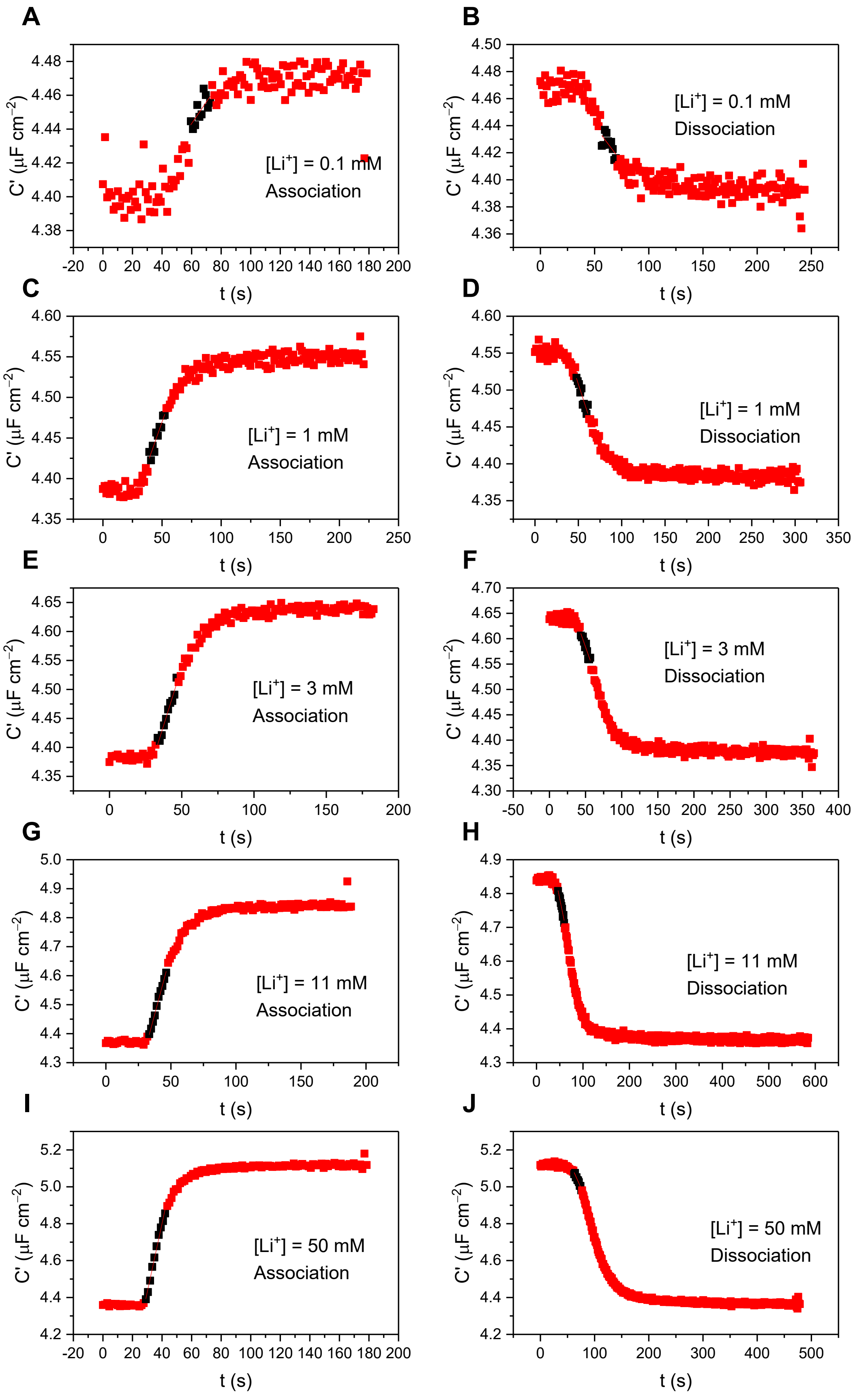

Figure S25. Association (left) and dissociation (right) traces, respectively, for a range of concentrations of Li^+: 0.1 mM (A and B), 1 mM (C and D), 3 mM (E and F), 11 mM (G and H) and 50 mM (I and J). Linear fits to the first 10 data points in the association/dissociation regime are shown in red.

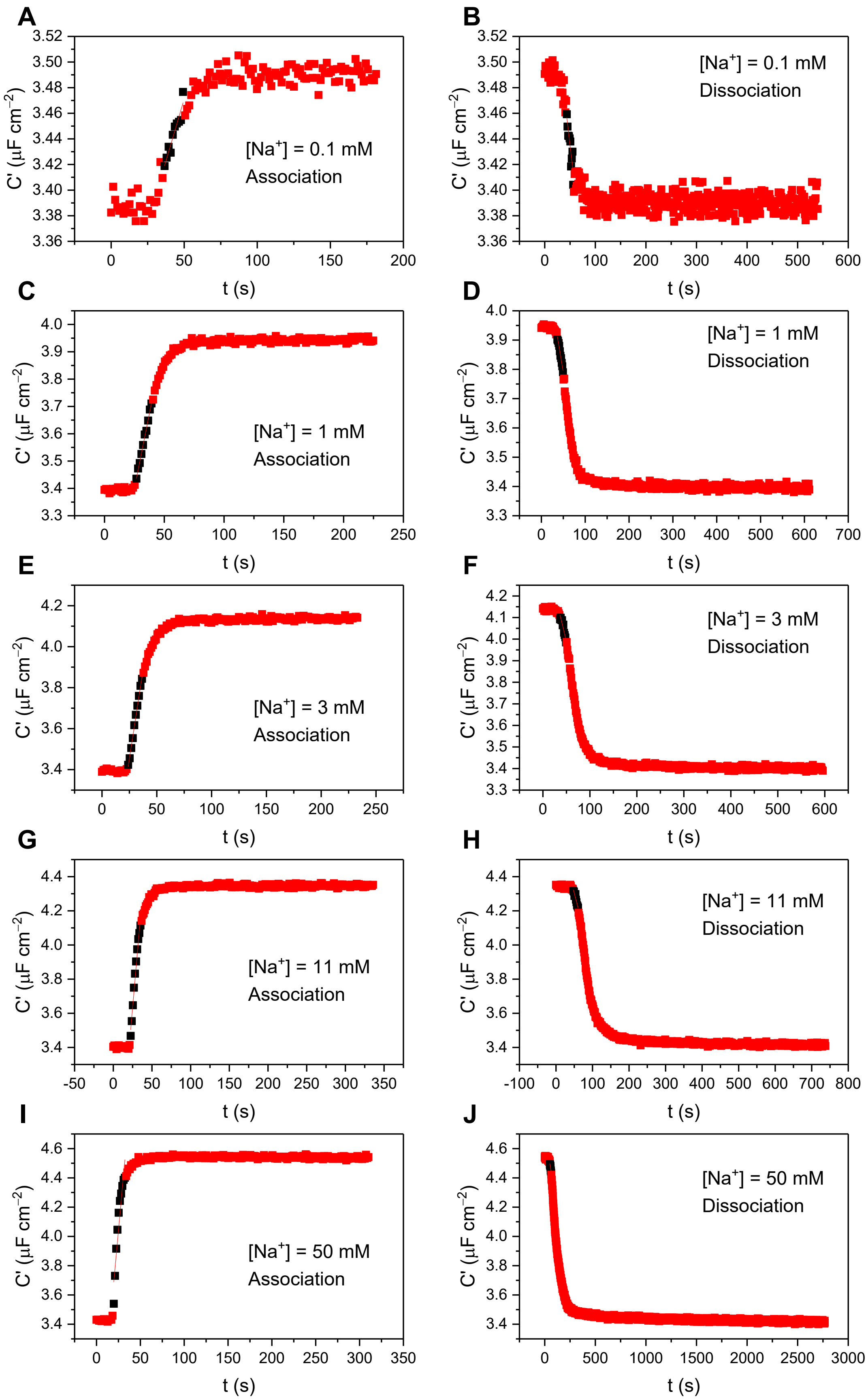

Figure S26. Association (left) and dissociation (right) traces, respectively, for a range of concentrations of Na^+: 0.1 mM (A and B), 1 mM (C and D), 3 mM (E and F), 11 mM (G and H) and 50 mM (I and J). Linear fits to the first 10 data points in the association/dissociation regime are shown in red.

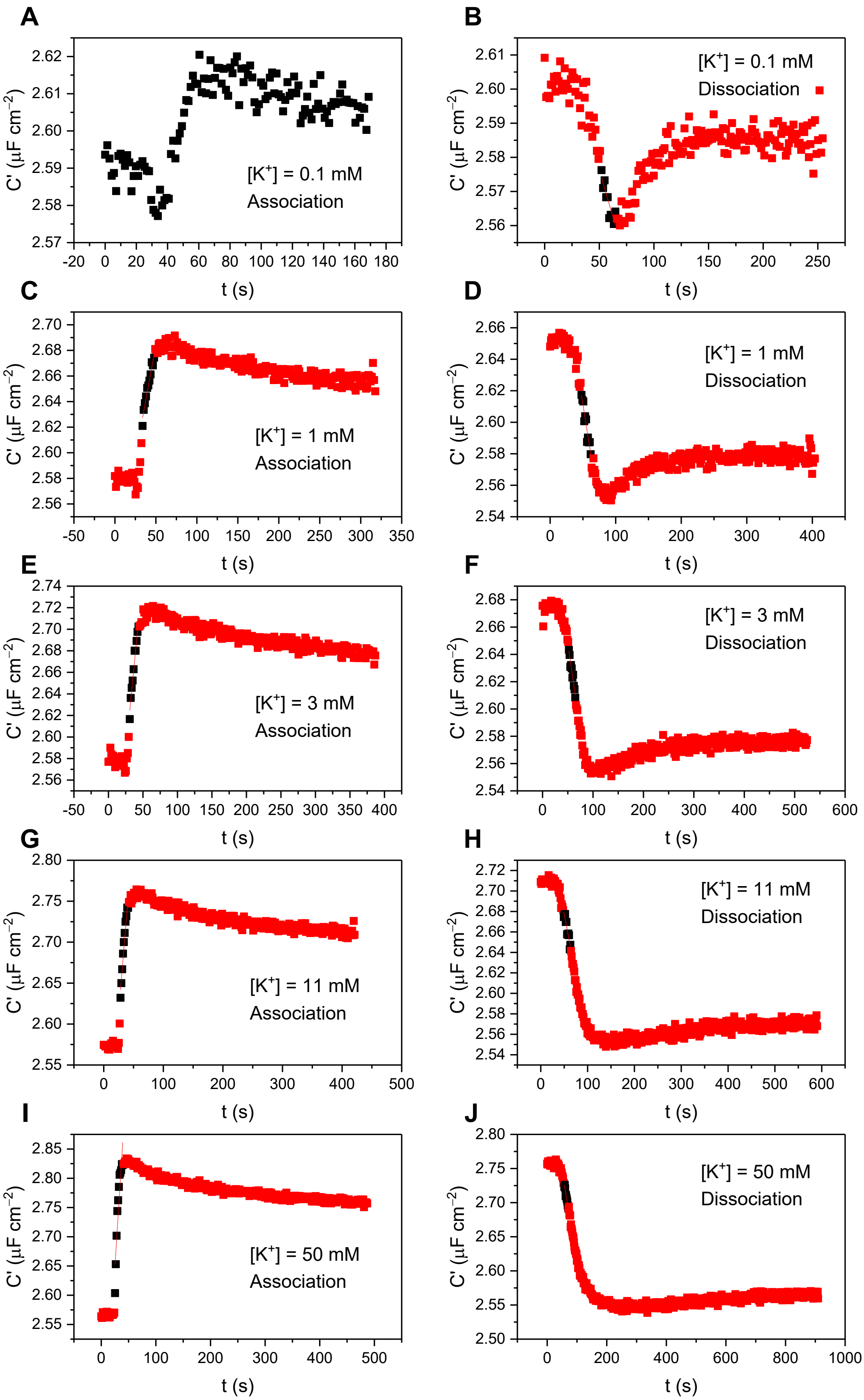

Figure S27. Association (left) and dissociation (right) traces, respectively, for a range of concentrations of K^+: 0.1 mM (A and B), 1 mM (C and D), 3 mM (E and F), 11 mM (G and H) and 50 mM (I and J). Linear fits to the first 10 data points in the association/dissociation regime are shown in red.

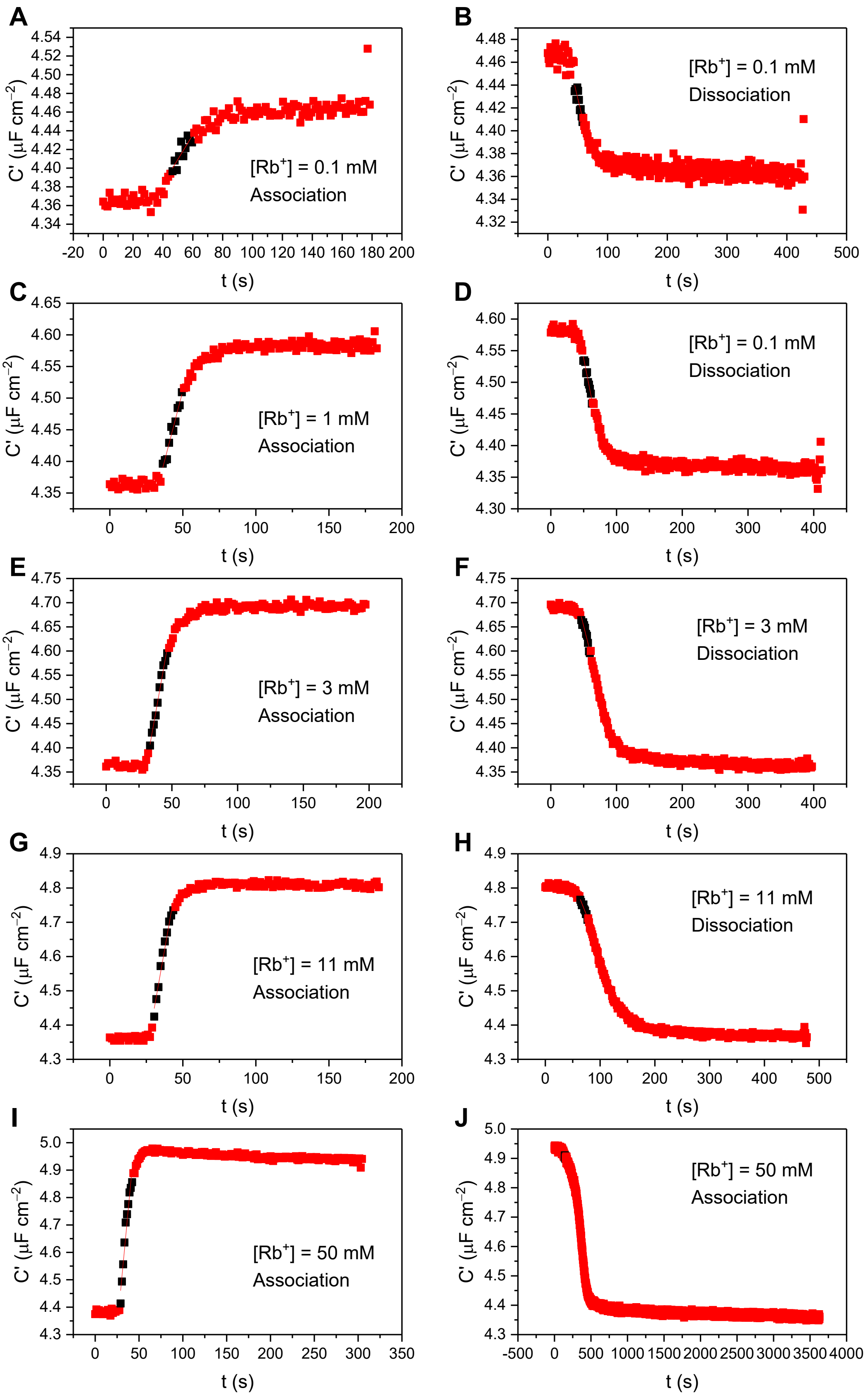

Figure S28. Association (left) and dissociation (right) traces, respectively, for a range of concentrations of Rb^+: 0.1 mM (A and B), 1 mM (C and D), 3 mM (E and F), 11 mM (G and H) and 50 mM (I and J). Linear fits to the first 10 data points in the association/dissociation regime are shown in red.

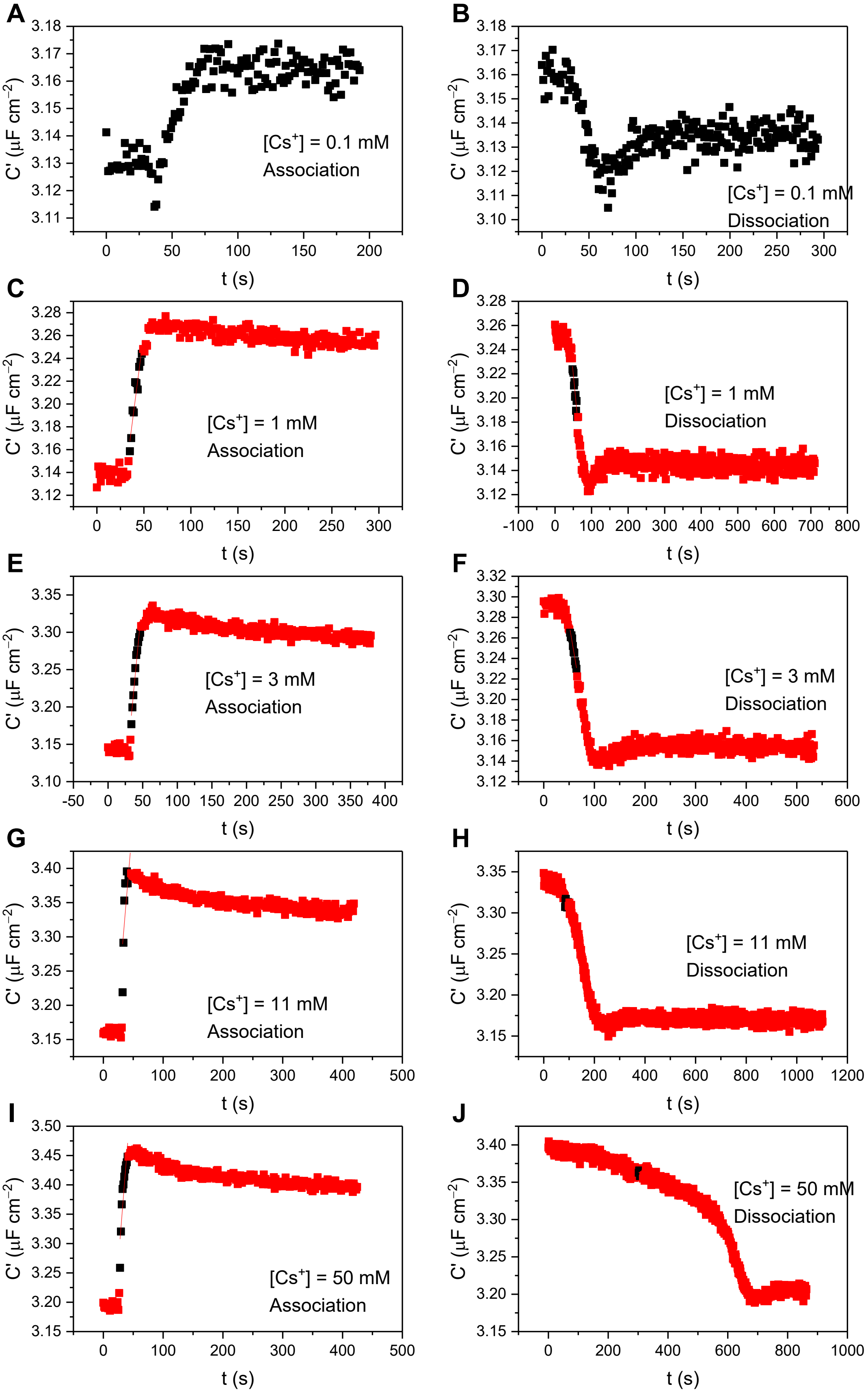

Figure S29. Association (left) and dissociation (right) traces, respectively, for a range of concentrations of Cs^+: 0.1 mM (A and B), 1 mM (C and D), 3 mM (E and F), 11 mM (G and H) and 50 mM (I and J). Linear fits to the first 10 data points in the association/dissociation regime are shown in red.

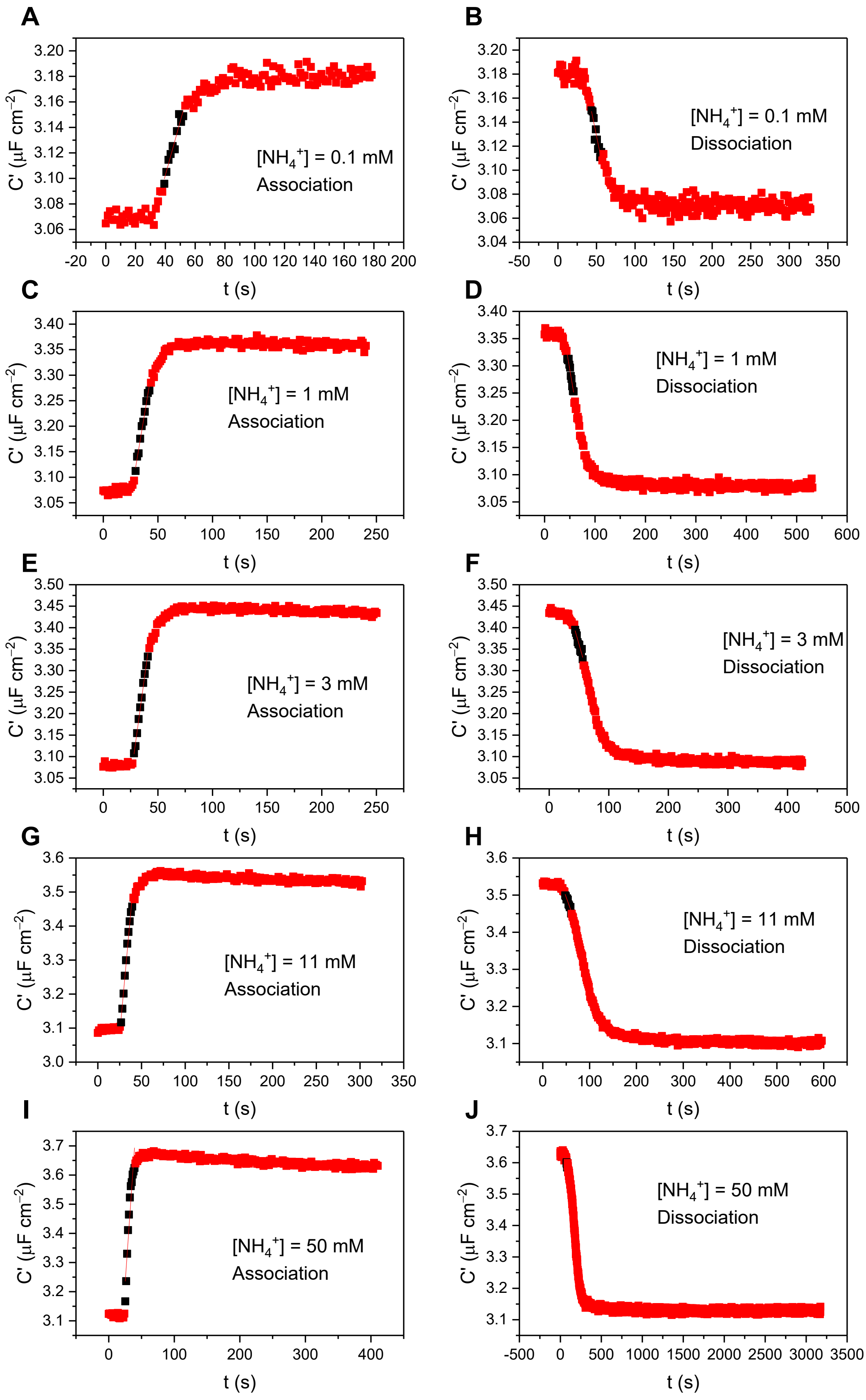

Figure S30. Association (left) and dissociation (right) traces, respectively, for a range of concentrations of NH_4^+: 0.1 mM (A and B), 1 mM (C and D), 3 mM (E and F), 11 mM (G and H) and 50 mM (I and J). Linear fits to the first 10 data points in the association/dissociation regime are shown in red.

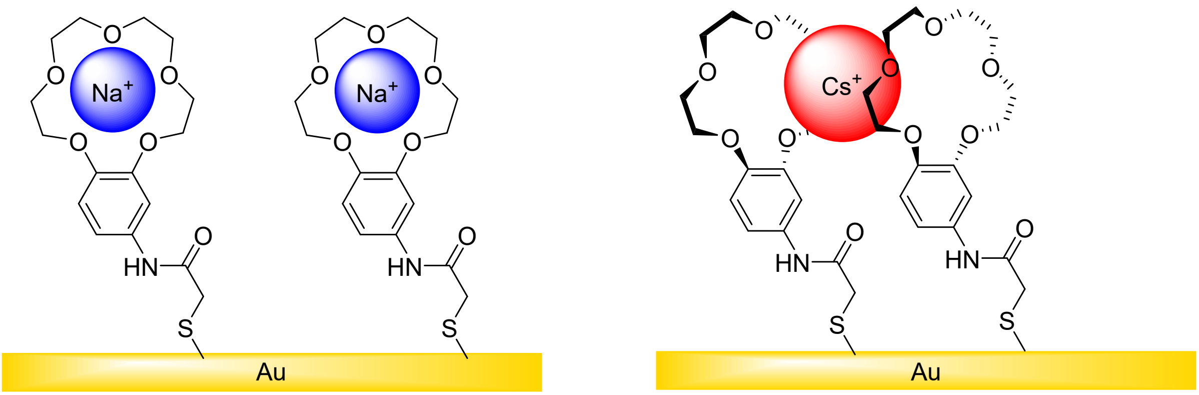

1:1 Binding Mode **B15C5$_{SAM}$** **2:1 Binding Mode**

Figure S31. Schematic depiction of 1:1 and 2:1 host-guest binding modes.

S9 References

1. C. M. Armstrong, *J. Gen. Physiol.*, 1971, **58**, 413-437.
2. R. M. Izatt, J. S. Bradshaw, S. A. Nielsen, J. D. Lamb, J. J. Christensen and D. Sen, *Chem. Rev.*, 1985, **85**, 271-339.
3. F. J. Millero, R. Feistel, D. G. Wright and T. J. McDougall, *Deep-Sea Res. Pt. I*, 2008, **55**, 50-72.
4. Z. Sonner, E. Wilder, J. Heikenfeld, G. Kasting, F. Beyette, D. Swaile, F. Sherman, J. Joyce, J. Hagen, N. Kelley-Loughnane and R. Naik, *Biomicrofluidics*, 2015, **9**.
5. S. C. Patrick, R. Hein, A. Docker, P. D. Beer and J. J. Davis, *Chem. Eur. J.*, 2021, **27**, 10201-10209.
6. R. Hein, A. Borissov, M. D. Smith, P. D. Beer and J. J. Davis, *Chem. Commun.*, 2019, **55**, 4849-4852.
7. S. C. Patrick, R. Hein, M. Sharafeldin, X. Li, P. D. Beer and J. J. Davis, *Chem. Eur. J.*, 2021, **27**, 17700-17706.
8. S. C. Patrick, R. Hein, P. D. Beer and J. J. Davis, *Journal of the American Chemical Society*, 2021, **143**, 19199-19206.
9. P. R. Bueno, R. Hein, A. Santos and J. J. Davis, *Phys. Chem. Chem. Phys.*, 2020, **22**, 3770-3774.
10. D. J. Cram, *Angew. Chem. Int. Ed.*, 1988, **27**, 1009-1020.
11. S. Flink, F. C. J. M. van Veggel and D. N. Reinhoudt, *J. Phys. Chem. B*, 1999, **103**, 6515-6520.
12. M. S. Góes, H. Rahman, J. Ryall, J. J. Davis and P. R. Bueno, *Langmuir*, 2012, **28**, 9689-9699.
13. J. Lehr, J. R. Weeks, A. Santos, G. T. Feliciano, M. I. G. Nicholson, J. J. Davis and P. R. Bueno, *Phys. Chem. Chem. Phys.*, 2017, **19**, 15098-15109.
14. S. C. Patrick, R. Hein, M. Sharafeldin, X. Li, P. D. Beer and J. J. Davis, *Chem. Eur. J.*, 2021, **27**, 17700-17706.
15. S. C. Patrick, R. Hein, P. D. Beer and J. J. Davis, *J. Am. Chem. Soc.*, 2021, **143**, 19199-19206.
16. A. Kumar, V. Kumar Tripathi, P. Sachan, A. Rakshit, R. M. Singh, S. K. Shukla, R. Pandey, A. Vishwakarma and K. C. Panda, in *Ecological Significance of River Ecosystems*, eds. S. Madhav, S. Kanhaiya, A. Srivastav, V. Singh and P. Singh, Elsevier, 2022, DOI: https://doi.org/10.1016/B978-0-323-85045-2.00011-X, pp. 187-202.
